# Genomic insights into the contribution of *de novo* lipogenesis to intramuscular fat deposition in chicken

**DOI:** 10.1016/j.jare.2023.12.003

**Published:** 2023-12-06

**Authors:** Huanxian Cui, Yongli Wang, Yuting Zhu, Xiaojing Liu, Lu Liu, Jie Wang, Xiaodong Tan, Yidong Wang, Siyuan Xing, Na Luo, Li Liu, Ranran Liu, Maiqing Zheng, Guiping Zhao, Jie Wen

**Affiliations:** State Key Laboratory of Animal Biotech Breeding, State Key Laboratory of Animal Nutrition and Feeding, Institute of Animal Science, Chinese Academy of Agricultural Sciences (CAAS), Beijing 100193, China

**Keywords:** Intramuscular fat, *FASN*, *de novo* lipogenesis, Myocyte, High-quality chicken meat

## Abstract

**Introduction:**

The proportion of animal based foods in daily diet of consumers is constantly increasing, with chicken being highly favored due to its high protein and low fat characteristics. The consumption of chicken around the world is steadily increasing. Intramuscular fat (IMF) is a key indicator affecting meat quality.

**Object:**

High IMF content can contribute to improve the quality of chicken meat. The regulatory mechanism of IMF deposition in chicken is poorly understood, so its complete elucidation is essential to improve chicken meat quality.

**Method:**

Here, we performed whole genome resequencing on 516 yellow feather chickens and single-cell RNA sequencing on 3 63-day-old female JXY chickens. In addition, transcriptome sequencing techniques were also performed on breast muscle tissue of JXY chickens at different developmental stages. And ^13^C isotope tracing technique was applied.

**Results:**

In this study, a large-scale genetic analysis of an IMF-selected population and a control population identified fatty acid synthase (*FASN*) as a key gene for improving IMF content. Also, contrary to conventional view, *de novo* lipogenesis (*DNL*) was deemed to be an important contributor to IMF deposition. As expected, further analyses by isotope tracing and other techniques, confirmed that *DNL* mainly occurs in myocytes, contributing about 40% of the total fatty acids through the regulation of FASN, using the available FAs as substrates. Additionally, we also identified a relevant causal mutation in the *FASN* gene with effects on FA composition.

**Conclusion:**

These findings contribute to the understanding of fat metabolism in muscle tissue of poultry, and provide the feasible strategy for the production of high-quality chicken meat.

## Graphical abstract

**Figure:**
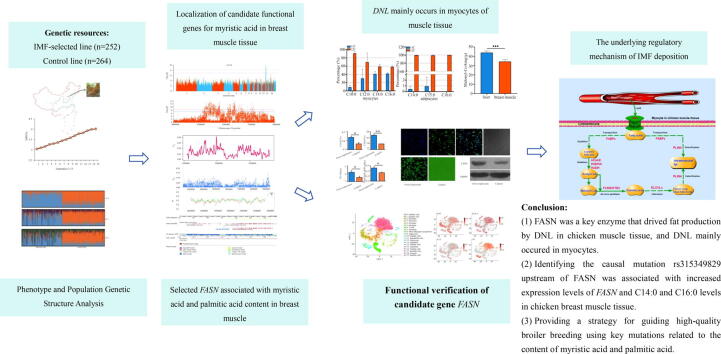

## Introduction

The improvement of the quality of meat products has always been a concern for meat producers. Although, increased fat deposition in muscle could induce glucose and lipid metabolism disorders in humans [1], [2], the increase of the intramuscular fat (IMF) content in meat is an effective measure of its quality, as IMF has an positive effects on livestock and poultry meat quality properties, such as tenderness, fragrance, etc. [3], [4]. For example, the excellent Kobe beef meat is known for its high IMF (also known as marbling) content.

However, the pursuit of growth speed and high-density feeding have resulted in a decrease in meat quality, due to the relatively higher water content and lower IMF content in meat [5]. Unlike marbling in beef cattle, most previous research mainly focused on the differential expression of some representative genes related to fat metabolism for the restriction of the unevenly distributed and imperceptible IMF in chicken [6], [7], [8]. At present, based on the Animal Quantitative Trait Loci (QTL) database (accessed in Nov 2023) [9], approximately 17 QTLs have been detected to be associated with the IMF percentage of chickens. In addition, based on high-density single nucleotide polymorphism detect associations of SNPs with IMF percent [10], [11], the mechanisms of action of these QTLs and SNPs are still unclear. Therefore, there has been no major breakthrough in the understanding of the genetic basis of IMF deposition in chicken meat.

Muscle tissue is mainly composed of myocytes, adipocytes, connective tissue cells, etc. [12], [13], [14]. IMF is a mixture of lipids deposited in muscle tissue through different pathways [15], [16]. Fatty acids (FAs) are not only important components of IMF in their free form, but also exist as a structural component as two main types of esters, namely triglycerides (TGs) and phospholipids (PLIPs) [17], [18]. Previous studies have found that the sources of FAs in chicken breast muscles involve two pathways: extracellular uptake and de novo fatty acid synthesis [19], [20]. Therefore, further research on FA metabolism in muscle tissue will also be important to fully elucidate the genetic basis of IMF deposition in chicken muscle tissue.

Since a proper experimental model is critical to the success of any research study, multi-omics analysis and further in vivo and in vitro studies on the genetics basis of IMF deposition were performed on an IMF-selected chicken population with increased IMF content and the appropriate control population. The goal in this study is to elucidate the molecular regulatory mechanism of IMF deposition in chicken muscle tissue. In addition, we anticipate that our data will provide theoretical support and also a feasible strategy for the improvement of meat quality and its production.

## Materials and methods

### Ethics statement

All procedures for the chickens used in this study were conducted in accordance with the guidelines for experimental animals developed by the Ministry of Science and Technology (Beijing, China). All experimental plans were approved by the Science Research Department of the Institute of Animal Sciences, Chinese Academy of Agricultural Sciences (Beijing, China), with reference number IAS2019-21.

### Animals and sample collection

A total of 516 female randomly chosen JXY chickens (n = 516; including 252 from an IMF-selected population and 264 from a control population) were obtained from the Institute of Animal Sciences, Chinese Academy of Agricultural Sciences (Beijing, China) for this study. The two populations originated from the same basic population of JXY100, which is a synthetic strain of chicken carrying the *dw* gene. The IMF-selected population was bred since the year 2000 for higher IMF content, and the control population was randomly bred. The IMF-selected population consisted of a total of 30 rooster families, and each generation was bred according to the male–female ratio of 1:3 to 1:4. The IMF content of each generation was determined as previously described [21].

The other 516 female chickens used in this study were from three Chinese native breeds (382 QYM, obtained from Guangdong Tinoo's Foods Co., Ltd., Qingyuan, China; 54 WC, obtained from Poultry Institute, Chinese Academy of Agricultural Sciences, Yangzhou, China; 80 E-JLH, obtained from Guangxi Jinling Agriculture and Animal Husbandry Group Co., Ltd., Nanning, China). All breeds of chickens are raised in three tiered cages (one chicken per cage) and fed with traditional corn-soybean meal diet. The blood samples of all chickens were collected with anticoagulant for genomic DNA (gDNA) extraction at 98 days of age. Subsequently, all chickens were euthanized by bleeding under carbon dioxide anesthesia. Then, we dissect and store the breast muscles, liver, and abdominal fat of each chicken at −20 ℃ or −80 ℃ for subsequent analysis. In addition, the breast muscle tissue of 3 63-day-old female JXY chickens was dissected for single-cell RNA sequencing analysis.

### Determination of TG content and fatty acid composition

The TG ELISA kit (Nanjing Jiancheng Bioengineering Institute, Nanjing, China) was used to homogenize the cell samples and quantify the results. To separate debris and fat, samples were homogenized at room temperature and centrifuged (1,000 × g, 20 min) at 4 °C. In order to optimize the accuracy, the assay was carried out in accordance with the manufacturer's instructions after dilution. Additionally, each of the three cell samples in 10-cm dishes was collected, freeze dried, and ground for FAs extraction and methylation using either the overexpression vector carrying the whole CDs of FASN or the empty vector. Then, the FA composition was measured by gas chromatography (GC) analysis in accordance with a previously described method [22].

### Whole-genome re-sequencing and Variant discovery and annotation

A total of 1,032 broiler chickens from four chicken breeds were used to extract gDNA samples from blood samples using the phenol–chloroform method (382 QYM, 54 WC, 80 E-JLH, and 516 JXY). The quality of gDNA was measured using NanoDrop-2000 spectrometer (Thermo Fisher Scientific Inc., Waltham, MA, USA) and agarose gel electrophoresis, respectively. For 516 JXY, a library of each DNA sample was constructed. All qualified libraries were sequenced using PE150 on the Illumina Novaseq platform by Beijing CapitalBio Technology Co., Ltd. (Beijing, China), with and each individual generated at least over 10 G of raw data. Raw data quality control and comparison refer to our previous published article [20]. Finally, based on the gene annotation of the reference genome (Gallus_gallus-6.0) (GCA_000002315.3) in Ensembl, the identified SNPs were annotated using ANNOVAR software [23]. (Accession number CRA002643 and CRA002650, https://bigd.big.ac.cn/gsa).

### Population structure analysis

The genetic data obtained from the IMF-selected population and the control population were evaluated, using the Admixture software (https://dalexander.github.io/admixture/) to analyze the group structure to determine the number of potential groups of JXY chickens.

### Genome-wide association study (GWAS) analysis for the content of FAs and IMF in JXY population

FAs is an important component of IMF. In order to elucidate the molecular regulatory mechanism of IMF deposition in chicken muscle tissue, GWAS of FAs and IMF was performed using the efficient mixed linear model (MLM) using the genome-wide efficient mixed model association (GEMMA) software [24]. Quality control analysis was conducted on genotype data (--mind 0.1, --maf 0.05) using PLINK v1.9 software [25]. A total of 9,614,458 SNPs were retained for GWAS after quality control. Considering the batch effect of the phenotype, the batch effect was added to the model as a fixed effect. The contents of FAs and IMF were determined by GWAS as follows:

Y=Xα+Zβ+Wμ+e

Y means phenotypic value, X means indicator matrix of fixed effect, Z means indicator matrix of SNP, W means indicator matrix for random effects, *α* means fixed effect, *β* means SNP effect, *μ* means random effects and *e* means residual item [26].

After quality control, the genetic association matrix was created on the SNPs, and the Wald test method was used to assess SNP significance. The significance threshold was determined by the Bonferroni multiple-testing correction. Whole-genome and suggestive significance threshold are determined by the Bonferroni multiple-testing correction (0.05/9,614,458, 1/9,614,458, respectively).

### Proportion of phenotypic variance explained (PVE) by SNPs

In this study, myristic acid content and genotype data from all 516 JXY chickens were used to the proportion of phenotypic variance explained (PVE) of SNP interpretation (equivalent to calculating the heritability of SNP). Use the marker correlation matrix (Gs) associated with phenotypic specific random effects of chicken myristic acid content across all genotypes to estimate PVE in GEMMA, which is a hard-called genotypes ($h^{2}=\sigma_{g}^{2}/\sigma_{p}^{2}$, i.e. genotypic variance (Vg)/ phenotypic variance (Vp)). The estimation of variance components was performed with the Genome-wide Complex Trait Analysis (GCTA) software [27], [28].

### Signature of selection analysis

Fixation index (F_ST_) analysis was conducted using the control group as the control group and the IMF selection group as the experimental group [29]. Specifically, the calculation of the F_ST_ value was based on the population differentiation principle, and the difference of allele frequency between two populations was compared using the VCFtools software [25]. We canned the entire genome with a 10 kb window size sliding and a 2 kb step size, and calculated the genetic differentiation index for each window. The top 1 % F_ST_ value was set as the significant threshold line, and the regions above the threshold line were defined as the selected region and gene annotation is performed.

### Target region and candidate gene identification

GWAS analysis was performed to detect associations between SNPs and the target gene or element, and identified the causative mutations and genes. The candidate regions were expanded to around ± 20 kb in size with the center regions beyond the chromosome threshold line to avoid the loss of genetic information. Additionally, the importance of candidate genes and SNPs were sorted according to the location. In addition, the obtained candidate regions by GWAS analysis and signature of selection were further integrated to define the overlapping region, as the genes and SNPs within the overlapping region might be important candidates associated with the target trait in chicken.

### Construction of reporter plasmids and detection of dual-luciferase reporter

The specific DNA fragment (2,000 base pairs) containing rs315349829[A] or rs315349829[G] were amplified by artificial synthesis and using site-directed mutagenesis. The products were used to generate the *FASN* promoter reporter plasmids with the site-specific mutation by cloning them into the pGL4.18 firefly luciferase expression vector. All cells were mycoplasma-free. 48-well plates containing 5 × 10^4^ 293 T cells were seeded for the dual luciferase reporter gene experiments. The RPMI-1640 medium containing 10 % fetal bovine serum (FBS, Gibco, Grand Island, NY, USA) was used for passage 2 of 293 T cells and maintained in a moist incubator at 37 ℃ and 5 % CO_2_. After cells had been transfected with allele reporter constructs for 48 h, their luciferase activity was measured using the Dual-Luciferase Reporter System (Promega Corporation, Madison, WI, USA). To bring the luciferase activity back to normal, the pBEC22 control vector encoding Renilla luciferase was also co-transfected. Three independent transfection experiments were performed for each plasmid design.

### RNA sequencing and data analysis

In this study, our previous RNA-sequencing analysis data of breast muscle tissues at different developmental stages was used (E12, E17, D1, D7, D21, D56, D98, D140, D180) (n = 3, total 24 birds), which was to study the expression profiles of candidate genes (Accession number CRA001334, http://bigd.big.ac. cn/gsa). In addition, we also used previous RNA sequencing data from breast muscle tissue samples from JXY chickens with high or low IMF content (n = 8, total 16 birds) (Accession number CRA001908). RNA was extracted from breast muscle tissue by using TRIzol reagent (Invitrogen, Carlsbad, CA, USA), and after the RNA was qualified, it was retroscribed and constructed from DNA and RNA sequencing was performed on Illumina NovaSeq 6000 S2 platform. The quality control data was compared with the reference genome [Ensembl GRCg6a (GCA_000002315.5)] to obtain gene expression levels.as previously reported [20].

### Weighted gene co-expression network analysis (WGCNA)

In order to further explore the correlation between gene expression and phenotype, and predict the function of genes in fat metabolism through gene co-expression, this study also conducted weighted gene co-expression network analysis (WGCNA) on RNA-seq data. WGCNA package was used to perform a weighted gene co-expression network analysis for all expressions of transcriptome sequencing data from 16 JXY chickens, and the (14,259) genes sequenced were correlated with phenotypes (FA composition, IMF and TG). This algorithm filters genes with the top 25 % variance as input data. The WGCNA was performed using the “WGCNA” package in the R software by constructing an adjacency matrix and a topological overlap matrix (TOM) and calculating the corresponding dissimilarity (1-TOM). Gene dendrogram construction and module identification were performed using a dynamic tree cut. WGCNA first loaded transcriptome data, erased missing values or outliers, and then loaded phenotypic information, visualized phenotypic data and gene expression data, selected an optimal soft threshold (β = 14) through scale-free distribution (R^2^ > 0.9) to build a co-expression network, identified modules significantly related to phenotypic data, and set the threshold for similar modules to merge 0.25. The hub genes are identified within the module with Gene significance > 0.2 and module membership > 0.8.Genotyping by MassArray® and genetic correlation analysis.

After determining the quality and quantity of gDNA samples of QYM, WC and E-JLH chickens, the MassArray detection was orderly performed using the MassARRAY® analyzer (Agena Bioscience, San Diego, CA, USA) by Beijing Compass Biotechnology Co., Ltd. (Beijing, China) to characterize the different genotypes of the candidate SNPs [30]. Briefly, after performing the specific polymerase chain reaction (PCR) amplification of the candidate SNPs on a Veriti® 384-Well Thermal Cycler (Applied Biosystems Inc., Foster City, CA, USA), the PCR product was treated with alkaline phosphatase. After completing the single base extension reaction and the resin purification, the PCR products were transferred on 384 chips for mass spectrometry detection. Eventually, the exported data were analyzed. The single SNP correlation analysis of the candidate SNP was performed in a total of 516 individuals from the other three population using the General Linear Model: y = Xα + Zβ + e (Xα: fixed effect, affecting the population structure of y, which is the breed factor; Zβ: marker effect; e: residual item) implemented in the PLINK software.

### Primary cells isolation and culture

In a sterile environment, the primary muscle satellite cells (MSCs) were isolated from the chest muscle tissue of 7-day-old chickens using the method previously reported[31]. Similarly, primary adipocytes were isolated from adipose tissue. The isolated MSCs and adipocytes were cultured in DMEM/F12 medium (Gibco, Grand Island, NY, USA) with 10 % FBS (Gibco, Grand Island, NY, USA) and 1 % Penicillin-Streptomycin (10,000 U/mL). Incubate in a humid atmosphere incubator at 37 ℃ and 5 % CO_2_. After the cell density reaches 90 %, the cells were digested with 0.25 % trypsin EDTA (Gibco Grand Island, NY, USA) for passage. After passage, the original cell density was maintained at 25 % − 30 % and continued to be cultured. The second-generation cells were collected after 48 h of cultivation for subsequent experiments.

### Vector construction and cell treatment

Based on the sequences of the chicken FASN gene in the NCBI and Ensembl GALGAL6.0 database (NM_205155.4 and ENSGALG00000040896), the expression vector carrying the complete coding sequence (CDS) of FASN was constructed. Then, the auxiliary plasmid Helper 1.0 and Helper 2.0 were used to obtain the lentivirus packaging vectors with a final titer of 2.5E + 8 TU/ml. The empty vector was also packeted as the control. The passage 2 MSCs were seeded at an initial density of 20 % or 30 % in 96-well plate or 10-cm dish and were respectively transfected with the overexpression vector carrying the complete CDS of *FASN* or the empty vector. After transfection, the contents of C14:0, C16:0 and TGs in cells were measured, and the green fluorescent protein were also observed under an electron microscope.

### Immunofluorescence

The MSCs were washed three times with PBS before being fixed in 4 % paraformaldehyde for 20 min, permeabilized in 0.2 % Triton X-100 for 15 min, and blocked with 10 % goat serum (CWBio, Beijing, China) for 30 min. The primary antibody FASN hybridoma supernatant (ab22759, 1:100; Abcam plc, Cambridge, UK) was then incubated with the cells for an overnight period at 4 °C. Following a thorough wash, the cells were incubated in the dark for 1 h with fluorescein isothiocyanate (FITC)-conjugated IgG (1:100, CWBio, Beijing, China). Using a Nikon Eclipse TE-2000-E Inverted Research Microscope (Nikon Solutions Co., Ltd., Tokyo, Japan), confocal fluorescence microscopy was used to detect and image MSC fluorescence signals in the bottom chambers.

### ^13^C Isotope Tracing Analysis

After primary myocytes and adipocytes attached, the culture medium (Gibco, Grand Island, NY, USA) containing 10 % FBS and 1 % Penicillin-Streptomycin (10,000 U/mL) was replaced with [U-13C] glucose (17.5 mM) (Sigma Aldrich, Shanghai, China) DMEM. The cells were collected after 96 h of incubation, and then 2 mL of precooled liquid mixture (water: methanol: chloroform volume ratio = 1:1:2) was added. The treatment was carried out in an ice-water bath for 5 cycles with the method of “1 min ultra-sonication and 1 min interval”. The chloroform layer extract was removed, centrifuged at 3500 × g and 4 ℃ for 10 min, and dried with nitrogen. Then the lipid extract was redissolved in 0.5 mL 75 % ethanol (containing 0.5 mM potassium hydroxide) in a water bath at 80 ℃. After cooling, 600 μL n-hexane was added, mixed evenly, and centrifuged for 10 min. Take all the supernatant and dry with nitrogen; Add 20 μL 1-hydroxybenzotriazole (HoBt), 40 μL Cholamine and 20 μL 2-(7-azabenzotriazol-1-yl)-N,N,N',N'-tetramethyluronium hexafluorophosphate (HATU), and derivate at room temperature for 10 min. Samples of equal volume were mixed from all prepared samples to obtain quality control samples. Reference to published articles [32] on the metabolic flow detection and analysis process of ultra-high performance liquid chromatography and high-resolution mass spectrometry. Export the data to obtain the original integrated area, and use reference methods to perform natural isotope correction on the original data [33].

### Determination of malonyl-CoA content

A chicken-specific ELISA kit (Enzyme-linked Biotechnology Co. Ltd., Shanghai, China) was used for measuring the amount of malonyl-CoA in the liver, myocytes, and adipocytes from muscle tissue, as well as the breast muscle tissue. Whirlpool oscillation was used to homogenize the cell samples at room temperature, and the debris and pellet were separated using a centrifuge (1,000 × g, 20 min) at 4 °C. As soon as possible, the supernatant was frozen at −80 °C for analysis. To maximize accuracy, the assay was carried out following the manufacturer's procedure and specified dilutions.

### Gene expression analysis of FASN

We used Quantitative real-time polymerase chain reaction (qPCR) to detect the *FASN* mRNA expression levels in myocytes and adipocytes and breast muscle tissue and liver tissue at different ages (E15, D1, D7, D35, D63, D98, D126), as well as in breast muscle tissue with different genotypes of *JXY* at 98 days of age. Total RNA was isolated from the samples using the TRIzol reagent (Invitrogen, Carlsbad, CA, USA) following the manufacturer’s protocol. The RNA concentration was determined on a Nanodrop-2000 spectrophotometer (Thermo Fisher Scientific Inc.). A total RNA of 2.0 μg was used in each sample for reverse transcription using the FastKing RT Kit (Tiangen, Beijing, China) according to the kit manufacturer's instructions to synthesize cDNA. The gene-specific primers of the *FASN* gene were designed based on Oligo 6.0 (Supplementary Table 1). Subsequently, cDNA, primers, double-distilled water and Green Master Mix were mixed to form a 10 ul system, with three replicates per sample for q-PCR. Reaction procedure: DNA was pre-denatured at 95 ℃ for 3 min, then denatured at 95 ℃ for 3 s and annealed at 60 ℃ for 34 s in 35 cycles. Expression was normalized to *ACTB* gene expression using the 2^−△△Ct^ method. The total protein of cells with the *FASN* over-expression and controls was extracted, and the protein concentration was measured with the bicinchoninic acid (BCA) protein assay kit. Protein samples were incubated with anti-FASN (1:500), Tubblin as the control protein.

10 × Single-cell RNA sequencing

The breast muscle tissue was removed from the cryopreservation solution and rinsed with pre-chilled Dulbecco’s phosphate-buffered saline containing 1 % FBS to remove the residual tissue protection solution, in a sterile environment. The enzymatic digestion separation of cells from the breast muscle tissue was performed according to a previously described protocol [31]. The prepared single-cell suspension is inspected first. After passing the quality inspection, Gel Beads and oil are added to different channels of Chromium Chip G respectively, and GEM (oil-in-water structure system) is formed through the microfluidic T cross system. One channel produces 80,000 to 100,000 oil droplets, 10 % of which contain cells and Gel Beads. Following cell lysis, Gel Beads automatically disintegrate and release a significant number of primer sequences. These primer sequences are then reverse-transcribed with mRNA that contains Poly A to produce the initial cDNA chain with a 10 *×* Barcode and unique molecular identifiers (UMIs) information. A strand of cDNA was isolated using magnetic beads following GEM fragmentation, and stable cDNA was produced by PCR amplification. The read2 sequencing primers were connected by terminal repair, addition of A, and splice to construct a 3-terminal expression profile library containing P5 and P7 splices and double-terminal index. Following this, the amplified cDNA was segmented by enzyme digestion, suitable length fragments were selected. Then, chose the paired-end program for double-ended sequencing, and Illumina’s data collecting software manages the sequencing procedure. After getting the original data, the Cell Ranger will first extract the barcode and UMI of the cells, then compare to the reference genome (Ensembl GRCg6a, ftp://ftp.ensembl.org/pub/release-100/gff3/gallus_gallus/) by STAR, correct the barcode, filter and correct UMI, and count. Then, according to the algorithm, barcode containing cells and background barcode were distinguished to extract the real single cell data and finally get the gene expression matrix of each cell.

### Cell types annotation and data analysis

We used Cellranger to transfer fastq files to the software of cell expression matrix, and calculated the filtering threshold that had to be set for each sample by calculating the distribution of UMIs, genes, mitochondria, and ribosomes of each sample. The normalized data were clustered using the Louvain algorithm [34], [35]. The T-SNE (t-distributed stochastic neighbor embedding) algorithm was used for data visualization analysis [36]. The Wilcoxon algorithm [37] was used to analyze the marker genes in all clusters, and the marker genes were scored in the way of group one vs. the rest. The genes with high specific expression in each cluster, logFC > 0.25 and expressed in at least 20 % of the cells were selected as the significant marker genes in the cluster. Using SingleR software and marker gene binding to annotate cell types [37]. The DEGs between the two types of cells were analyzed using the edgeR package of the R software. The gene expression fold change > 1.5 or < 0.67 and P value < 0.05 were identified as DEGs. The pathway enrichment analysis to identify significantly enriched signaling pathways of DEGs were performed on the Kyoto Encyclopedia of Genes and Genomes (KEGG) pathway database using Kobas 3.0 [38].

### Statistical analyses

The phenotypic correlation was calculated by Spearman correlation analysis using the SPSS 22.0 software, and the correlation Heatmap was plotted by the Heatmap.2 package in the R language gplots. The box plot of the SNP combination for the myristic acid content phenotype was generated by the “ggpubr” package in the R software. Using GraphPad software (Prism version 9.4.1) for student t-tests to test the significance of differences between different groups. Data are expressed as the mean ± standard deviation (SD).

## Results

### Genes related to fatty acid metabolism involved in IMF deposition

**Fig. 1:**
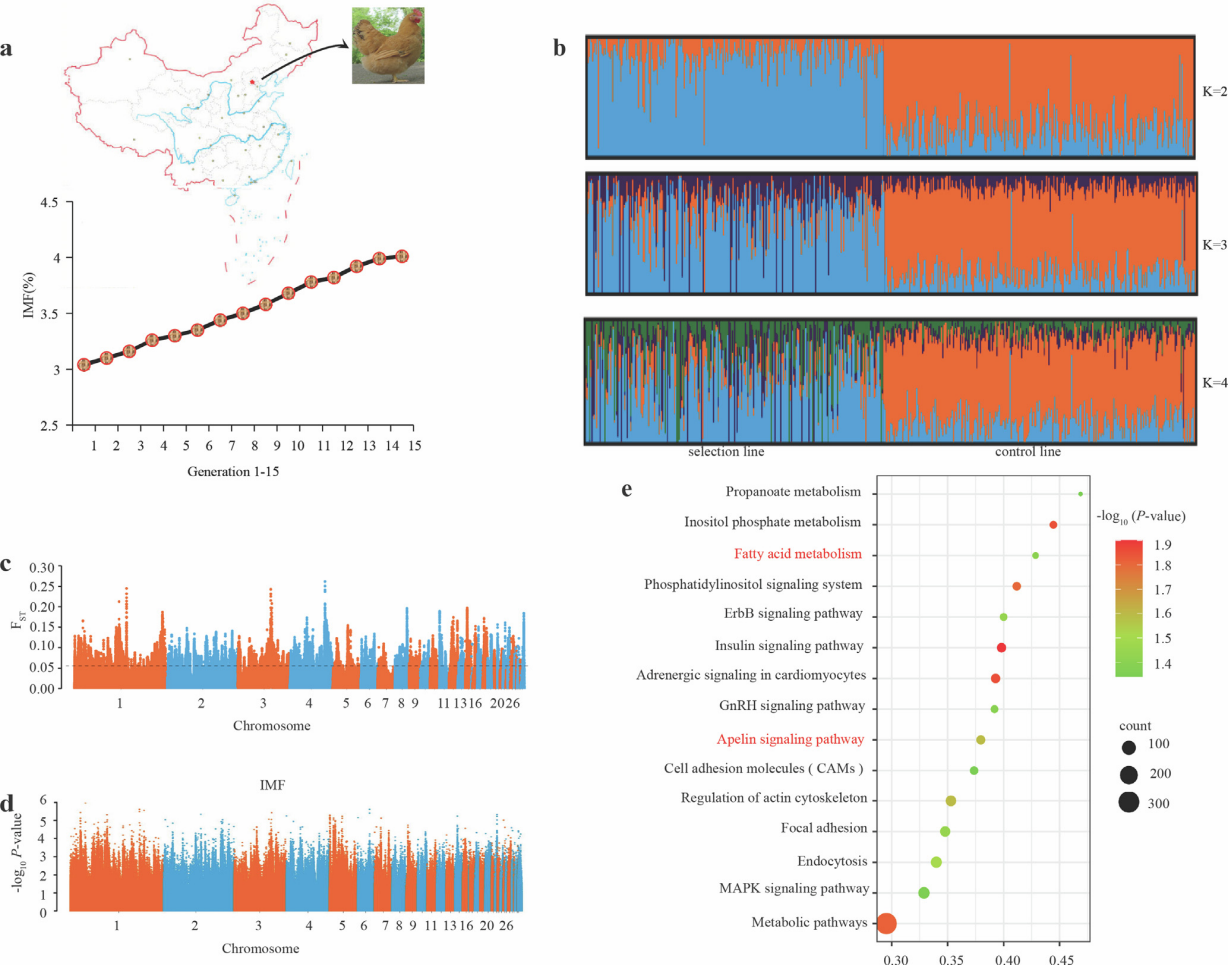
Genome-wide identification of genes associated with IMF deposition. a. The content of IMF in breast muscle tissue from 1 to 15 generations of the IMF-selected lines showed a continuous increasing trend. b. The population structure analysis of the IMF-selected and the control populations (K = 2, 3, 4, respectively), showing the separation of the two populations. c. The significant selection signature by the fixation index (*F_ST_*) according to the top 1 % threshold, with a noticeable genetic differentiation (n = 516). The black dashed line represents the top 1 % threshold line. d. GWAS analysis of the IMF content at the genome-wide significance threshold (−log10 *p* = 8.28) or suggestive threshold (−log10 *p* = 6.98), no genome-wide loci associated with the IMF content (n = 516). e. The enriched signaling pathway based on 3,981 annotated genes by the top 1 % SNPs (n = 98,869).

We found that the IMF content in breast muscle tissue of the selected chicken population increased from generations 1 to 15 (Fig. 1a). Then, chickens from the IMF-selected chicken population with significantly increased IMF content at generation 16 and the control population were re-sequenced. Based on a total of 9,614,458 single nucleotide polymorphisms (SNPs) after quality control, the IMF-selected and the control populations were divided into two clusters by population structure analysis (Fig. 1b). Additionally, a significant selection signature detected by the fixation index (F_ST_) according to the top 1 % threshold (Fig. 1c, Supplementary Table 2) was found in multiple chromosomes, containing 845 annotated genes (Supplementary Table 3). This indicated that phenotypic selection leads to genomic differentiation. In addition, after pathway enrichment analysis and screening some significantly enriched lipid metabolism-related pathways were identified, including the glycerolipid metabolism, glycerophospholipid metabolism, PPAR, and adipocytokine signaling pathways (Supplementary Fig. 1, Supplementary Table 4). After investigating the genetic basis of IMF deposition through a statistical analysis of the phenotypic data, 516 individuals (including 252 chickens from the IMF-selected population and 264 chickens from the control chicken population) were chosen for a GWAS on IMF content (Supplementary Table 5). However, we found no genome-wide loci significantly associated with IMF content at the genome-wide significance threshold (−log_10_ p = 8.28) or suggestive threshold (−log_10_ p = 6.98) (Fig. 1d). Instead, we identified candidate genes controlling IMF content using the top 1 % SNPs (n = 98,869) in the whole-genome (Supplementary Table 6). Based on the 3,981 annotated genes found (Supplementary Table 7), the FA metabolism and Apelin signaling pathway were identified as significantly enriched signaling pathway related to lipid metabolism (highlighted in Fig. 1e). Among them, the FA metabolism pathway is mainly involved in FA biosynthesis (catalyzed by enzymes encoded by ACLY, ME, ACACA, and FASN, etc.), carbon chain extension (including ELOVL fatty acid elongase 5 encoded by ELOVL5, etc.), acylation (including enzymes encoded by ACSL3, ACSL4, etc.) and oxidation (including enzymes encoded by ACADL, etc.) (Supplementary Fig. 2).

### Selected FASN and rs315349829 associated with myristic acid and palmitic acid content in breast muscle

**Fig. 2:**
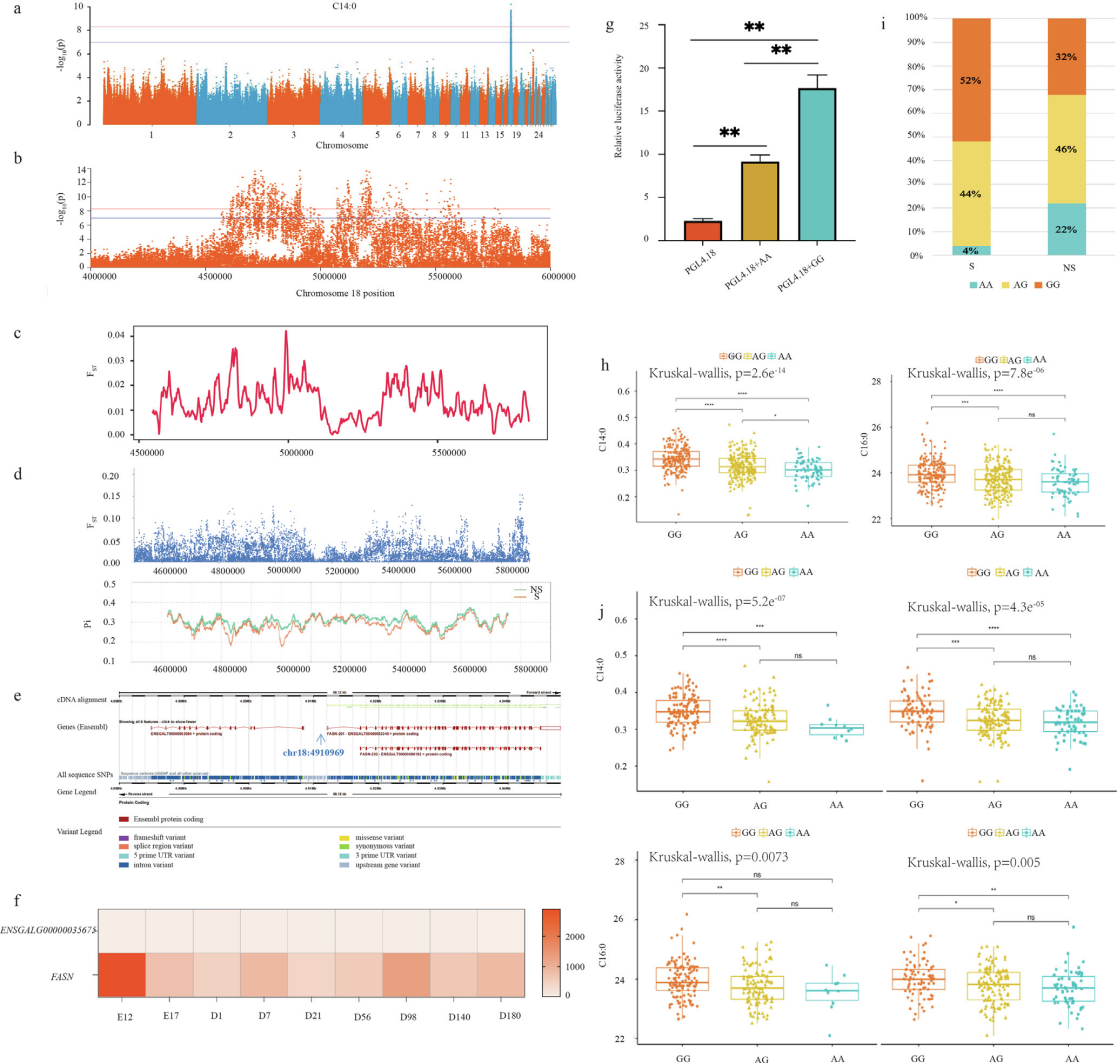
Identification of genetic variations related to fatty acid content in breast muscle. a. Manhattan plot for myristic acid content in JXY chicken. The red and blue lines represent the genomic and suggestive thresholds. b. After expanding the region to 1.251 Mb (chr18:4,548,962–5,800,023) with the suggestive threshold line ± 20 kb in chromosome 18. c. The selective sweep by *F_ST_* with a 10-kb window at the chr18:4,548,962–5,800,023 in chromosome 18, only a weakly selected region was found. d. The selection signal of single site by *F_ST_* (the standard value with 0.1) and Pi at the chr18:4,548,962–5,800,023 in chromosome 18. e. The annotated genes by five identified SNPs with the association to C14:0 content. f. Expression levels of *FASN* and *ENSGALG00000035675* in breast muscle tissue g. The regulation of different homozygous genotypes ([G] and [A] alleles) of the SNP rs315349829 on the *FASN* promoter expression (n = 3 cells repeats). h. The C14:0 and C16:0 contents in breast muscle tissue of JXY homozygotes carrying the [GG] or [AA] of the SNP rs315349829 (n = 516). i. Distribution of the number of different genotypes of the SNP rs315349829 in the IMF-selected population and the control population of JXY chicken (n = 516). j. The C14:0 and C16:0 contents in breast muscle tissue of JXY homozygotes carrying the [GG] or [AA] of the SNP rs315349829 in the selected population (n = 252) and control population (n = 264). *p < 0.05, **p < 0.01, ***p < 0.001. (For interpretation of the references to colour in this figure legend, the reader is referred to the web version of this article.)

Considering the above results and the important contribution of FAs to IMF deposition[19], we performed a GWAS on FA composition (Supplementary Table 8), and found a locus on chromosome 18 significantly associated with the myristic acid (C14:0, the intermediate products of de novo synthesis of FAs) content in breast muscle tissue (Fig. 2a and 2b), which is also associated with the palmitic acid content (C16:0, the end products of de novo synthesis of FAs) (Supplementary Fig. 3). To avoid the omission of useful information, we expanded the region to 1.251 Mb (chr18:4,548,962–5,800,023) with a suggestive threshold line of ± 20 kb, containing the 14,938 SNPs (Supplementary Table 9), with a higher and stronger linkage relationship (Supplementary Fig. 4). For significantly higher contents of myristic acid and palmitic acid in the selected population than those in the control population (Supplementary Fig. 5), we performed a selective sweep by F_ST_ with a 10-kb window, but only found a weakly selected region (Fig. 2c). Our investigation of the genetic selection signal of a single site in this region, by measuring the F_ST_ (the standard value with 0.1) and Pi (Fig. 2d, Supplementary Table 10), identified five SNPs associated with C14:0 content, located in the upstream non-coding region of FASN (one SNP) and ENSGALG00000035675 (four SNPs) (Fig. 2e). According to our reported transcriptome data of JXY chickens[39], the FASN gene is expressed in breast muscle tissue, but not ENSGALG00000035675 (Fig. 2f). Thus, the SNP (chr18:4910969, rs315349829 [A/G]) present in the upstream of FASN was found to be tightly linked with the highest SNP of significant region (chr18:4910989, rs312544499 [G/A]) at a distance of 20 bp (Supplementary Fig. 6). A dual-luciferase reporter assay by separately transfecting individual pGL4.18 vectors carrying the corresponding genes revealed that the [G] allele of rs315349829 resulted in significantly higher FASN promoter activity than that the [A] allele (Fig. 2g). We also found that the C14:0 and C16:0 contents in breast muscle tissue of 516 chickens carrying the alternate (alt)-genotype [GG] of rs315349829 were significantly higher than those in homozygotes carrying the reference (ref)-genotype [AA] (Fig. 2h), showing the enhancing effect of rs315349829 on the biosynthesis of C14:0 and C16:0 in breast muscle tissue of JXY chickens. In addition, we also investigated the effect of rs315349829 on other FAs and found that the contents of C14:1, C16:1, C20:1 and C21:0, except for C14:0, C16:0, were significantly higher in the alt-genotype [GG] homozygotes than in the ref-genotype [AA] (Supplementary Fig.7). Furthermore, the estimated phenotypic variance indicated that the SNP rs315349829 could account for 27.74 % of the contribution to myristic acid content of the total genome-wide genetic variations in breast muscle tissue of JXY chickens (Supplementary Table 11).

The number of GG and AG individuals with higher C14:0 and C16:0 contents were higher in the IMF-selected population than that in the control JXY chicken population, while the number of AA homozygotes with lower C14:0 and C16:0 contents was lower in the IMF-selected population than that in the control chicken population (Fig. 2i and 2j), which might explain the higher C14:0 and C16:0 contents in the IMF-selected population. Moreover, the analysis of the rs315349829 distribution showed that this mutation was widespread in chicken breeds from all over the world as an [A] to [G] mutation (Supplementary Fig. 8). Thus, we also investigated the relationship of rs315349829 with the C14:0 content in the breast muscle tissue of 516 individual chickens from other breeds, namely Wenchang chicken (WC), Qingyuan ma chicken (QYM) and E line of Jinling huang chicken (E-JLH) (Supplementary Table 12), and found significant correlation for C14:0 content (r = 0.687, P = 1.55E-06) and C16:0 (r = 0.687, P = 1.55E-06) (Supplementary Table 13), which supported the wide mutational effect of rs315349829 on the regulation of the C14:0 and C16:0 contents in the muscle tissue of multiple breeds.

### DNL mainly occurs in myocytes of muscle tissue

**Fig. 3:**
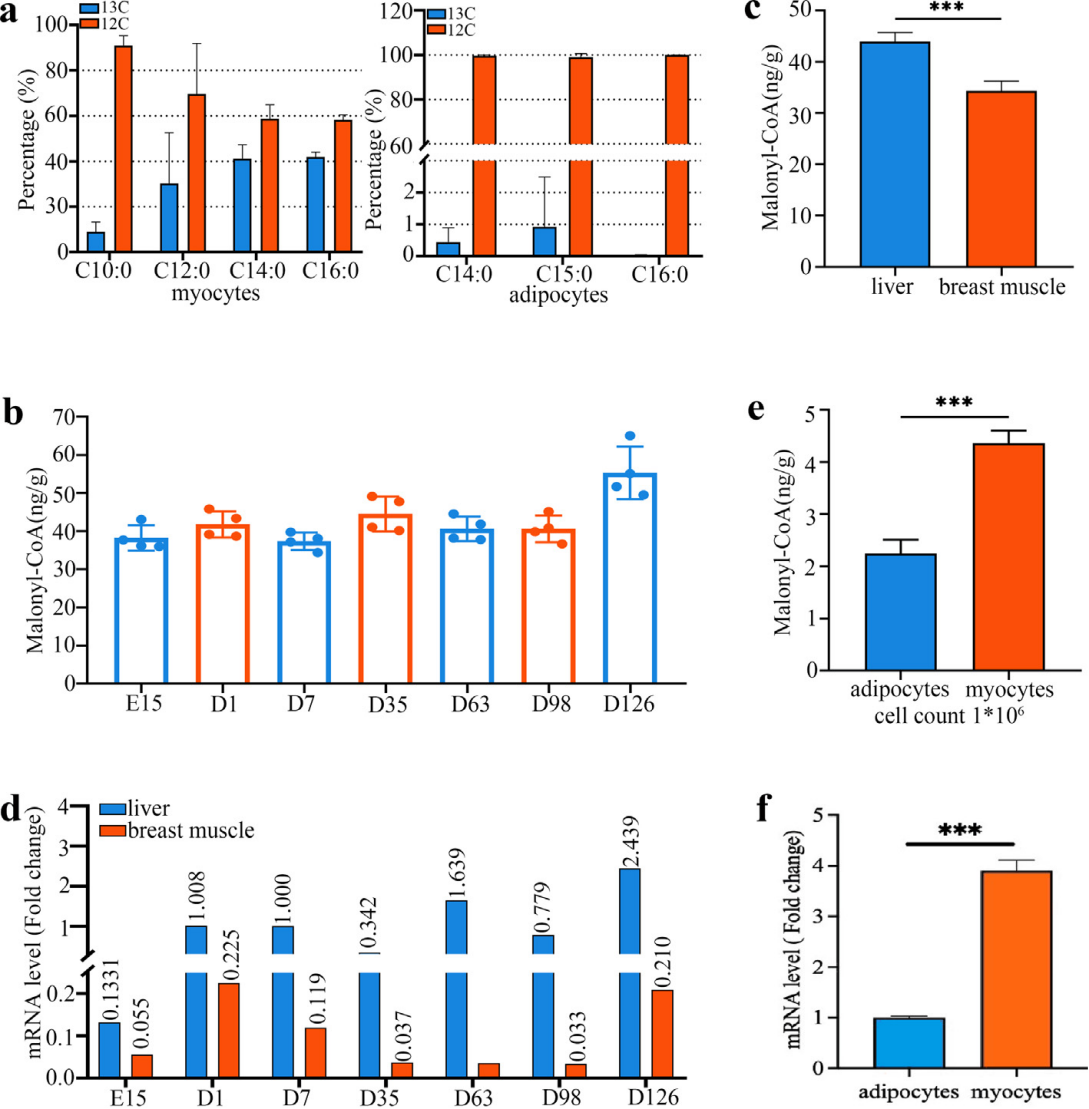
Occurrence of *DNL* in breast muscle tissue of chicken. a. The in vitro detection of *de novo* FAs with the radioisotope-label in both myocytes and adipocytes from the breast muscle tissue (n = 3 cell repeats). b. Content of malonyl-CoA in muscle tissue at all the different developmental stages (n = 28). c. and d. Comparisons of malonyl-CoA content and *FASN* mRNA level between the muscle tissue and the liver (n = 28). e. and f. Comparisons of malonyl-CoA content and *FASN* mRNA level in myocytes and adipocytes from muscle tissue. *p < 0.05, **p < 0.01, ***p < 0.001.

Having determined the effect of FASN on *DNL*, we further investigated whether *DNL* occurs in muscle tissue of chicken by studying the metabolism and distribution of FAs with techniques using radioisotope-labelled FAs. As expected, the isotope-labelled FAs were found in both myocytes and adipocytes after culturing for 48 h with radioisotope-labelled glucose as the starting substrate (Fig. 3a). Furthermore, it was also found that the proportion of isotope-labelled FAs in myocytes was about 35 %−40 % of the total FAs, while the proportion of FAs labeled in adipocytes was very small. In addition, we also measured the content of malonyl-CoA, an irreversible first product of *DNL*. First, malonyl-CoA was found in muscle tissue at all the different developmental stages (Fig. 3b), indicating the common occurrence of *DNL* in chicken muscle tissue. To our surprise, a further comparison revealed that the level of malonyl-CoA in muscle tissue was about 80 % of that in the liver (Fig. 3c), with similar pattern observed in the *FASN* mRNA level (Fig. 3d). Additionally, we also performed in vitro measurements of malonyl-CoA content and *FASN* mRNA level in myocytes and adipocytes from muscle tissue, and surprisingly found that both the malonyl-CoA content and *FASN* mRNA level in myocytes were significantly (*P* < 0.05) higher than those in adipocytes (Fig. 3e).

### FASN promotes IMF deposition through DNL

**Fig. 4:**
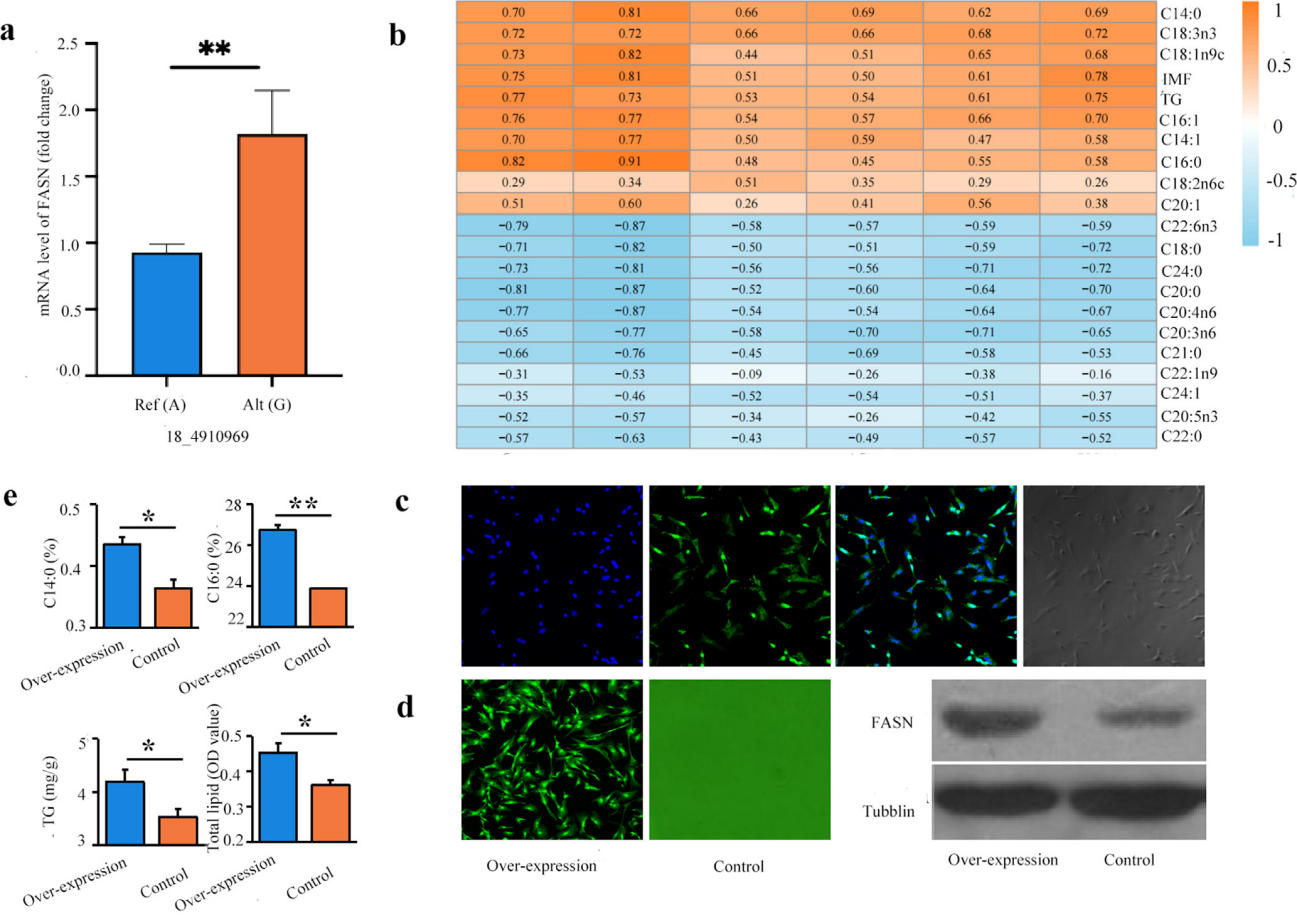
Effect of FASN on IMF deposition through DNL. a. The expression level of FASN in breast muscle tissue of homozygous carrying GG or AA of rs315349829 (n = 12). b. The relationship between the expression levels of FASN and representative genes related to lipid metabolism co-expressed with FASN and the contents of FAs and TG in breast muscle tissue (n = 16). The relationship between expression level of FASN and FAs, TG contents in breast muscle tissue by WGCNA (n = 16) and the representative genes related to lipid metabolism with the genes co-expressed with FASN. c-e. The changes of C14:0 and TG contents with the increase of FASN expression level (n = 3 cells repeat). *p < 0.05, **p < 0.01, ***p < 0.001.

To verify the function of the FASN protein encoded by the FASN gene in the production of IMF by DNL, more experiments were performed. As expected, gene expression analysis revealed that the expression levels of FASN in GG homozygous were significantly higher than those in AA homozygous (Fig. 4a), which is consistent with the higher correlation of the FA content and IMF/TG content in breast muscle tissue of chickens previously reported [17]. We also performed a WGCNA on the genes expressed in breast muscle tissue (n = 14,259) by transcriptome and phenotypic data (including IMF, TGs and FAs) (Supplementary Fig. 9a, Supplementary Table 14). Gene modules were obtained by differential hierarchical clustering analysis, and a total of 22 modules were identified after merging (Supplementary Fig. 9b). The FASN gene was present among the genes in the significantly enriched ME light yellow module, which is associated with the IMF, TG, C14:0, C14:1, C16:0, C16:1, C18:1n9c, C18:3n3 and C20:1 trait. The relationship revealed a significant correlation between the FASN mRNA level and TG content (r = 0.526, P = 0.044), C14:0 (r = 0.658, P = 0.0076), as shown is Fig. 4b. Additionally, a total of 521 representative genes related to lipid metabolism that were considered as the co-expressed genes of FASN were found in the ME light yellow module (Supplementary Table 15), mainly involved in FA metabolism (ACLY and ACSS1), TG lipolysis (DGKA and LPL) and esterification (PLIN4). In addition, we further investigated the regulatory effect of FASN on DNL in myocytes from the chicken breast muscle tissue by analyzing the overexpression of FASN, and found that the contents of C14:0 and TG were significantly increased (Fig. 4c) with the increase of the FASN level (Fig. 4d and 4e).

### FASN is widely expressed in various types of cells in chicken muscle tissue

**Fig. 5:**
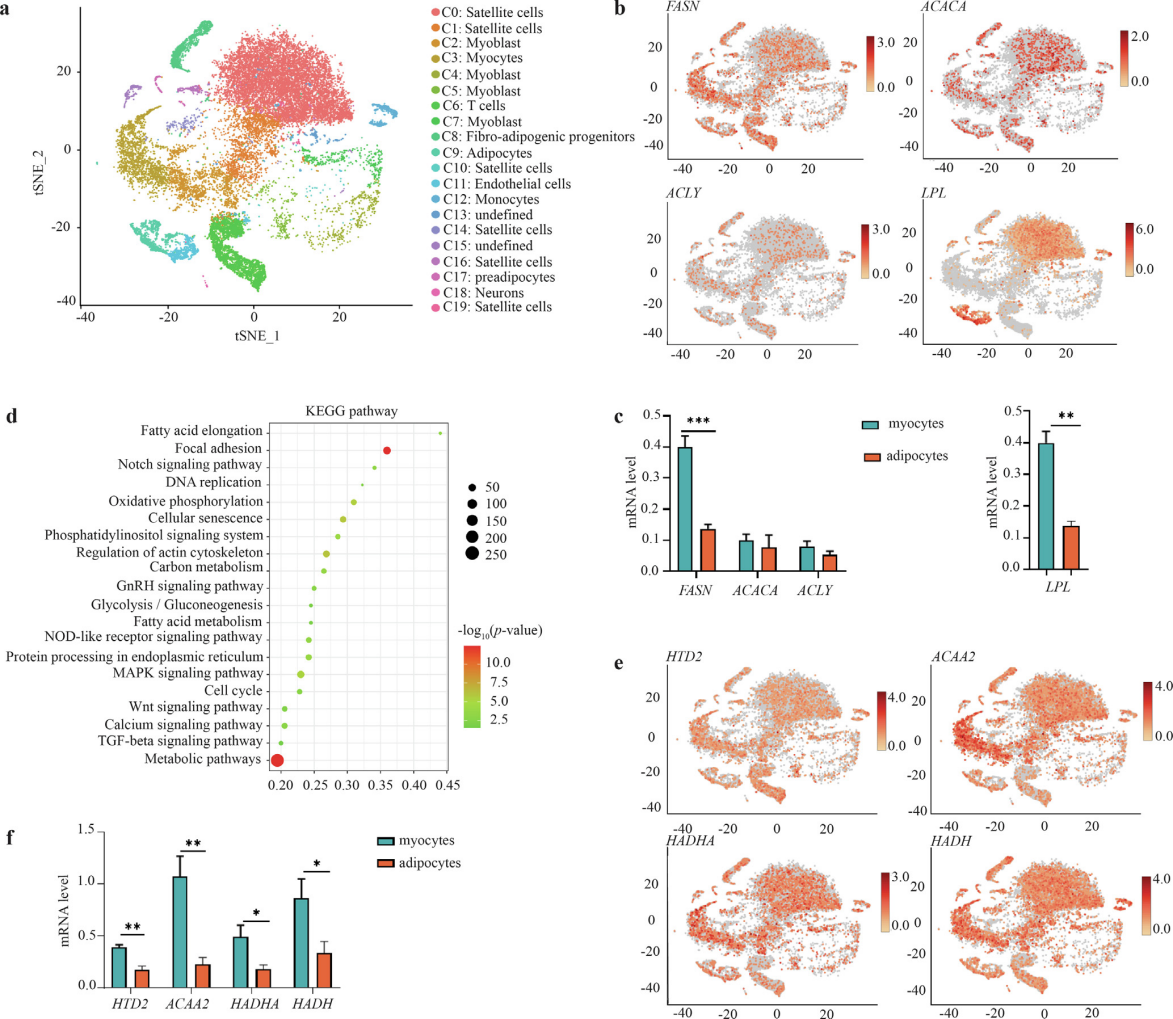
The key regulation of FASN in *DNL* in muscle tissue. a. The 20 identified cell subsets in the breast muscle tissue by single-cell sequencing, mainly including a muscle cell population and adipocytes with 68.12 % and 10.37 %. b. and c. The expression levels of representative genes related to the *DNL* (*FASN* and *ACACA*) and lipolysis (*e.g*., *LPL*) in different cell types, and the upregulation of *FASN* expression and the downregulation of *LPL* expression in myocytes compared to adipocytes from breast muscle tissue (n = 3). d. The enrichment of the FA metabolism pathway by KEGG pathway enrichment analysis based on 3,483 DEGs. e and f. Expression levels of *HTD2*, *ACAA2*, *HADHA*, *HADH* except for *FASN* related to the FA metabolism in different cell types*.* *p < 0.05, **p < 0.01, ***p < 0.001.

We performed single-cell RNA sequencing to more accurately establish the cytological basis of *DNL*. The cell activity in three chicken breast muscle samples at the age of 63 days is shown in Supplementary Table 16. The single sample filtering threshold used was a proportion of mitochondrial gene expression of less than 20 %, and the number of cell-expressed genes was 200–5,000. Detailed statistical results, such as median genes per cell, unique molecular identifiers (UMIs), Q30 bases in barcode and valid barcodes, are shown in Supplementary Table 17 and Supplementary Fig.10. A total of 20 cell subsets were systematically identified by the marker genes referencing to the database of other species, including muscle cell population, adipocytes, monocytes, T cells, neurons, *etc*. (Fig. 5a). Among them, the populations related to myocytes and adipocytes accounted for about 68.12 % and 10.37 %, respectively. Furthermore, we measured the expression levels of some representative genes in different cell types (Fig. 5b), and found that the genes involved in the *DNL* (*FASN*, *ACACA*) were expressed in all the cell types, and had their stronger signal in the muscle cell population. Similarly, the genes involved in the lipolysis (*e.g*., *LPL*) were expressed in all the cell types.

### FASN was still the key gene regulating the DNL in muscle tissue

For the above detected difference in *DNL* levels between myocytes and adipocytes in breast muscle tissue, we also performed a comparative analysis of the differentially expressed genes (DEGs, myocytes vs. adipocytes), and a total of 3,483 DEGs (fold change > 1.5 or < 0.67, *P* value < 0.05 were identified (Supplementary Table 18), including 1,591 upregulated genes and 1,892 downregulated genes. It is worth noting that in myocytes the expression level of *FASN* was significantly upregulated, while that of *LPL* was significantly downregulated compared to those in adipocytes (Fig. 5c). In addition, KEGG pathway enrichment analysis of 3,483 DEGs showed the FA metabolism as a key significantly enriched pathway (Fig. 5d), mediated the proteins encoded by *FASN*, *HTD2*, *ACAA2*, *HADHA*, *HADH*, *etc*. (Fig. 5e and 5f). In the FA metabolism pathway, the *de novo* synthesis of FAs is catalyzed by the enzyme encoded by the *FASN* gene using acetyl-CoA or malonyl-CoA from FA oxidation as substrate, and is extended into various very-long chain FAs (Supplementary Fig.11).

## Discussion

Poultry is the largest produced and consumed meat worldwide, its consumption is determined by its deliciousness, tenderness and juicy quality. It has been established that high IMF content is an important factor contributing to meat flavor, texture and other desirable properties [40], [41], [42], thus increasing the IMF content represents an effective approach to improve meat quality [43], [44]. However, the genetic basis of IMF production in chicken remains unclear. Therefore, to gain a better understanding of the genetic basis of IMF production and elucidate the mechanism of IMF deposition in chicken, we performed a systematic study using an experimental model with a phenotype of high IMF content. This study not only revealed that FASN, the gene encoding the multienzyme protein responsible for the conversion of glucose into FAs by *DNL*, is the major gene promoting IMF deposition in chicken, but also that the SNP rs315349829 may be the causal mutation responsible for the increase of C14:0, C16:0 and TG contents in muscle tissue, which will be useful to guide the breeding strategy for high-quality chicken meat.

A GWAS analysis of the IMF content in chicken breast muscle tissue revealed that IMF is a complex trait regulated by multiple genes and gene networks involving genes related to FA metabolism, which is supported by the RNA-sequencing and single-cell sequencing data. These findings were also consistent with our previously reported results [20]. We then focused on elucidating the genetic basis of the trait of IMF content in chicken by investigating FA metabolism in breast muscle tissue using a multi-omics combination approach with undeniable advantages in the systematic screening of genetic variations in key functional genes associated with quantitative traits [45], [46]. We found the SNP rs315349829 in the upstream region of *FASN*, which may be responsible for the increase of the C14:0 and C16:0 contents by upregulating the expression level of FASN in chicken breast muscle tissue. These results suggested that the SNP rs315349829 and *FASN* gene could be responsible for the increase in the IMF content in the breast muscle of the IMF-selected chicken population compared to the control population. Given that C14:0 and C16:0 are the intermediate and end products, respectively, of FA de novo biosynthesis [47], and *FASN* encodes the key enzyme driving *DNL* [48], [49], we hypothesized that *DNL* might be important in promoting IMF deposition in chicken as suggested by our previous studies. However, the prevailing theory holds that in poultry *DNL* mainly occurs in the liver, and little to none is thought to occur in muscle tissue and adipose tissue [50], [51]. Therefore, to confirm our hypothesis on the contribution of *DNL* to IMF deposition, we performed additional in vivo and in vitro isotope tracing (using a gold-labeling method) experiments that detected the presence of malonyl-CoA (the irreversible first intermediate in the synthesis of FAs by *DNL*) and the occurrence of de novo synthesis of FA in IMF deposition in chicken.

**Fig. 6:**
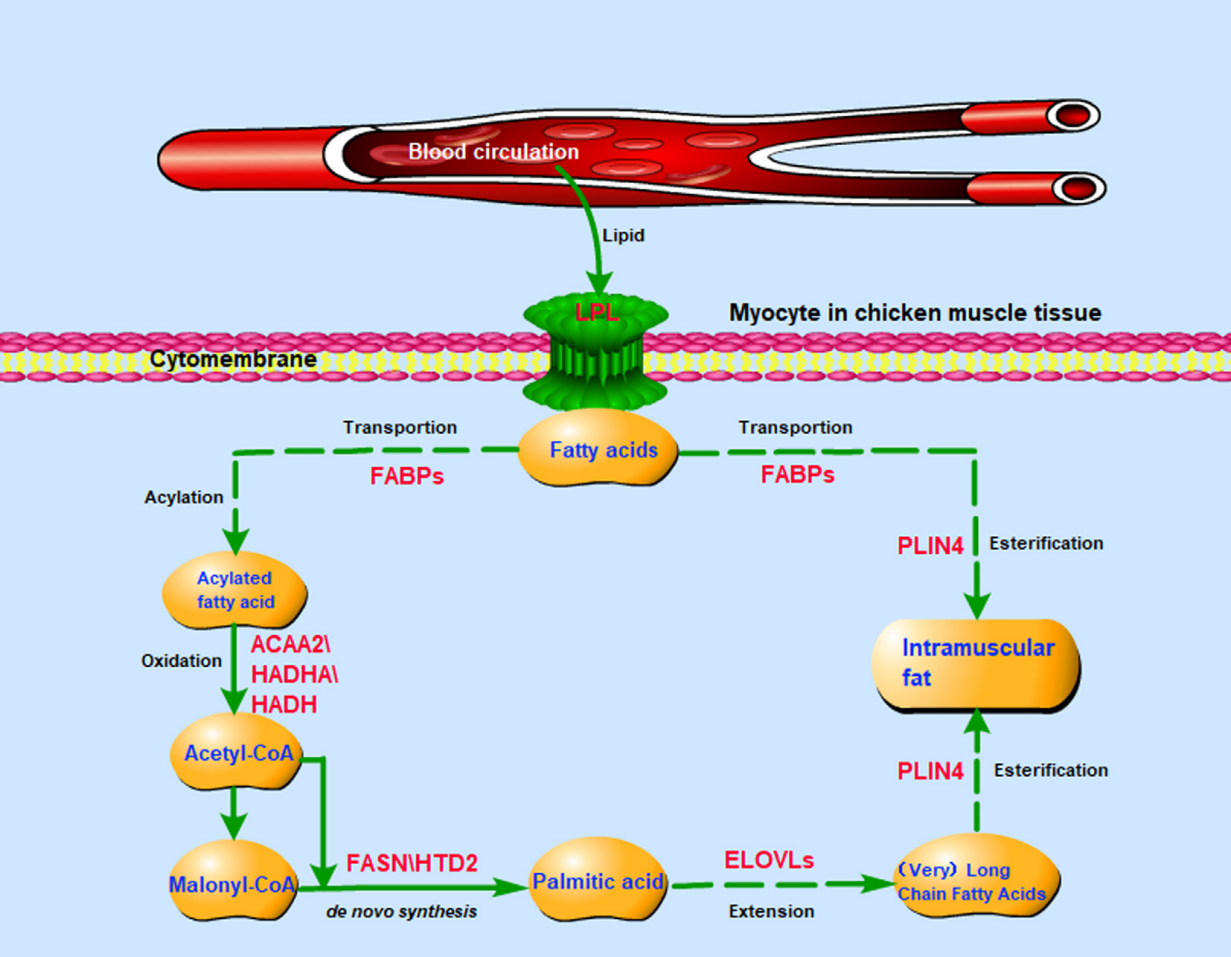
The underlying regulatory mechanism of IMF deposition in chicken muscle tissue. Muscle tissue uses the energy produced by the oxidation of FA obtained through FA cellular uptake (ACAA2, HADHA and HADH), and the excess energy is used to regenerate FA by *DNL* (FASN and HTD2).

It is generally believed that most IMF is stored in adipocytes [52], not in myocytes, except when ectopic deposition occurs under pathological conditions [53]. Surprisingly, although the IMF content in chicken muscle tissue has been reported to be low (only 2 %−4 %), the proportion of newly synthesized isotope-labelled FAs was determined to be 35 %−40 % of the total FAs in myocytes. Therefore, our results support that *DNL* occurs in both myocytes and adipocytes from muscle tissue, but mainly in myocytes, consistent with the physiological characteristics of myocytes, whose energy needs are mainly met by oxidative degradation (catabolism) of FAs obtained by cellular uptake and the excess energy could be stored nearby [54], [55]. Our data obtained using single-cell sequencing technology [56], [57], revealed that the FASN gene was most highly expressed in the muscle cell groups, again confirming the occurrence of *DNL* in chicken muscle tissue. As expected, the results revealed that the FA metabolism pathway was enriched based on the difference in *DNL* FA production between myocytes and adipocytes, and FASN promoted *DNL* using the acetyl-CoA or malonyl-CoA as substrate. Moreover, additional enzymes encoded by other identified DEGs (*ACAA2*, *HADHA*and *HADH*) promote the non-de novo synthesis of myristic acid that is degraded into acetyl-CoA to provide the starting substrate for *DNL*. In other words, muscle tissue uses the energy produced by the oxidation of FAs obtained by FA cellular uptake, and the excess energy is used to regenerate FAs by *DNL*. Based on all the above results, we proposed the underlying regulatory mechanism of IMF deposition in muscle tissue of chicken (Fig. 6).

## Conclusion

In conclusion, this study reports elucidates the molecular mechanism of increased IMF content in chicken muscle tissue. Our results revealed the important contribution of *DNL* to IMF deposition with FASN as the crucial enzyme driving fat production by *DNL* in chicken muscle tissue, which occurs mainly in myocytes. This study also identified the causal mutation rs315349829 upstream of *FASN*, which is associated with C14:0 and C16:0 content in chicken breast muscle tissue by increasing the expression level of FASN. These findings enrich our insights into fat metabolism in poultry muscle tissue. and provide a strategy using a key mutation associated with myristic acid and palmitic acid content to guide the breeding of high-quality meat chicken.

## Compliance with ethics requirements

The care and use of all individuals in the experiment comply with all Institutional and National Guidelines.

## CRediT authorship contribution statement

**Huanxian Cui:** Conceptualization, Methodology, Software, Data curation, Visualization, Writing – review & editing. **Yongli Wang:** Conceptualization, Methodology, Data curation, Visualization, Writing – review & editing. **Yuting Zhu:** Conceptualization, Data curation, Visualization, Writing – review & editing. **Xiaojing Liu:** Conceptualization, Data curation, Writing – review & editing. **Lu Liu:** Conceptualization, Data curation, Writing – review & editing. **Jie Wang:** Investigation, Software. **Xiaodong Tan:** Investigation, Software. **Yidong Wang:** Investigation, Software. **Siyuan Xing:** Investigation, Software. **Na Luo:** Investigation, Software. **Li Liu:** Investigation. **Ranran Liu:** Investigation. **Maiqing Zheng:** Investigation. **Guiping Zhao:** Conceptualization, Methodology, Writing – review & editing. **Jie Wen:** Conceptualization, Methodology, Writing – review & editing.

## Declaration of Competing Interest

The authors declare that they have no known competing financial interests or personal relationships that could have appeared to influence the work reported in this paper.
