# Supplementary material for: Genomic insights into the contribution of de novo lipogenesis to intramuscular fat deposition in chicken
Source: J Adv Res. 2023 Dec 6;65:19–31. doi: 10.1016/j.jare.2023.12.003 (PMC11519054; doi:10.1016/j.jare.2023.12.003)
Supplement: Supplementary data 1 [file mmc1.docx]

**Supplementary Information for**

**Genomic Insights into the Contribution of *De Novo* Lipogenesis to Intramuscular Fat Deposition in Chicken**

Huanxian Cui^†^, Yongli Wang^†^, Yuting Zhu^†^, Xiaojing Liu^†^, Lu Liu^†^, Jie Wang, Xiaodong Tan, Yidong Wang, Siyuan Xing, Na Luo, Li Liu, Ranran Liu, Maiqing Zheng, Guiping Zhao^*^, Jie Wen^*^

State Key Laboratory of Animal Biotech Breeding; State Key Laboratory of Animal Nutrition and Feeding; Institute of Animal Science, Chinese Academy of Agricultural Sciences (CAAS), Beijing 100193, China

^†^ These authors contributed equally to this work.

^*^ Corresponding authors: Guiping Zhao, E-mail: zhaoguiping@caas.cn; Jie Wen, E-mail: wenjie@caas.cn

**This file includes:**

Supplementary Figs S1 to S11

Supplementary Tables S1 to S18 (see the excel files)

**Supplementary Figures**


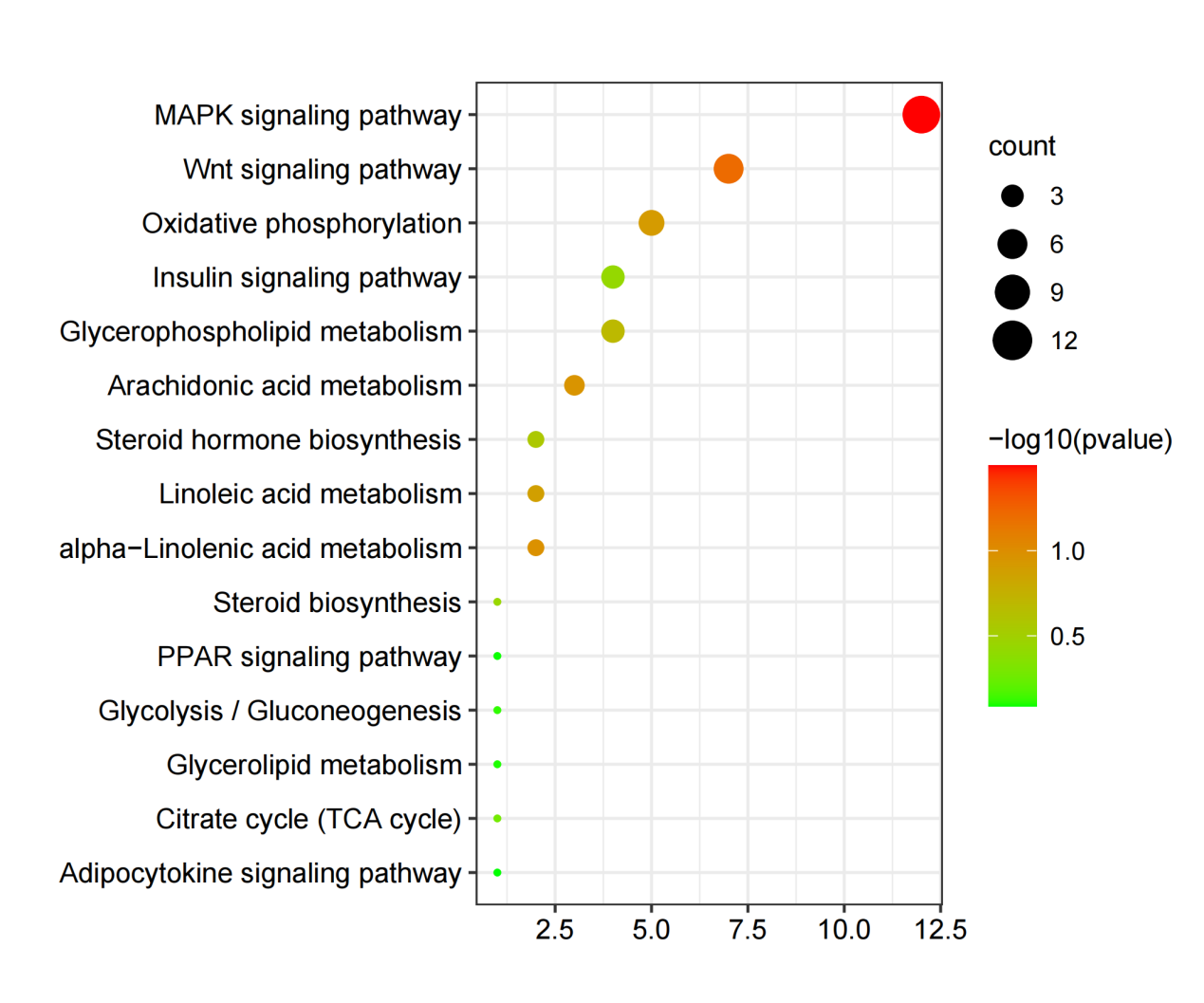


**Figure S1:** The enriched pathways by KEGG pathway enrichment analysis based on 845 annotated genes for identifying the significant selection signature with the top 1% threshold by *F_ST_*.

**
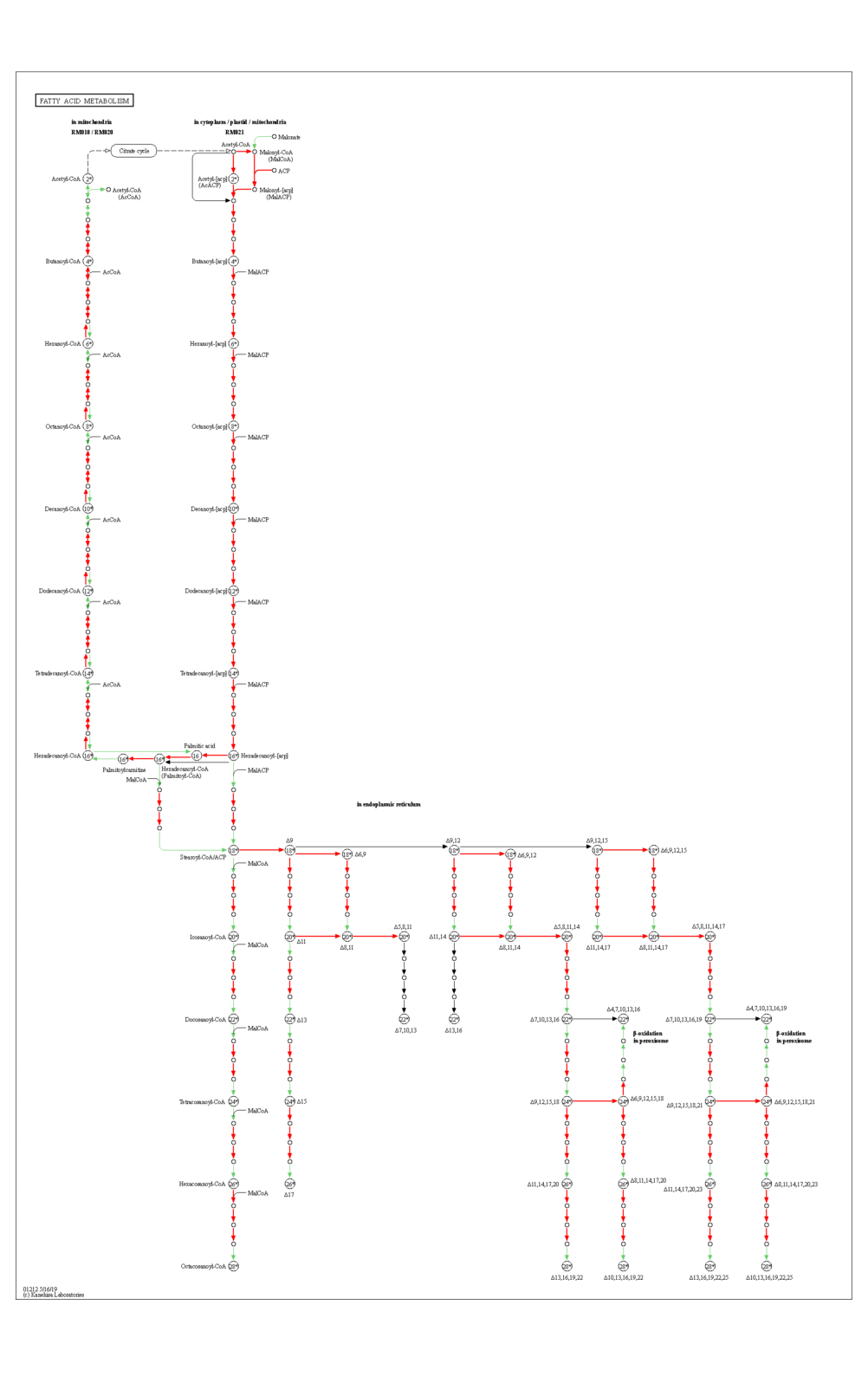
**

**Figure S1:** The enriched FA metabolism pathway based on the genes identified by GWAS analysis associated with IMF content in breast muscle tissue, involved in FA biosynthesis, carbon chain extension, acylation and oxidation.

**
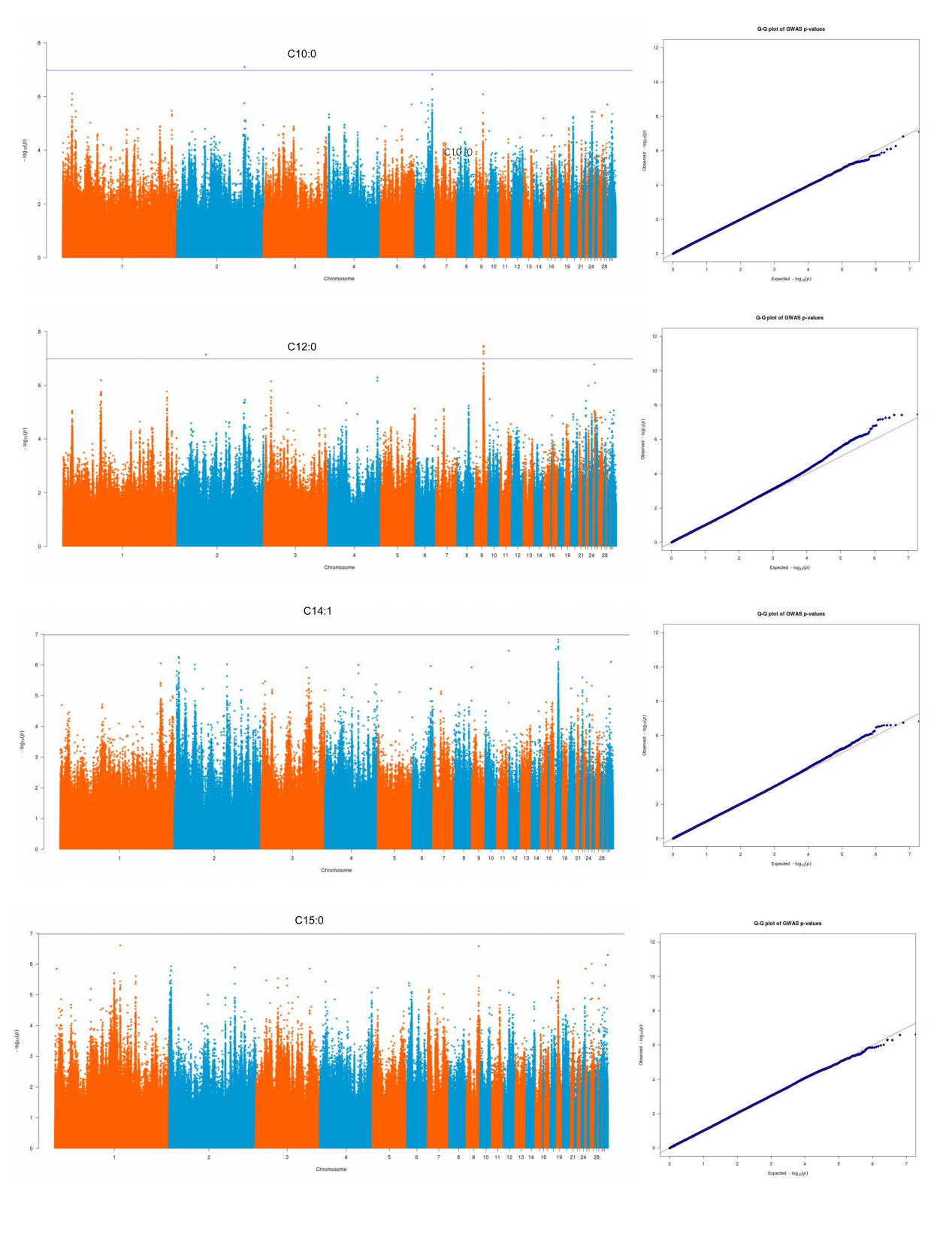
**

**
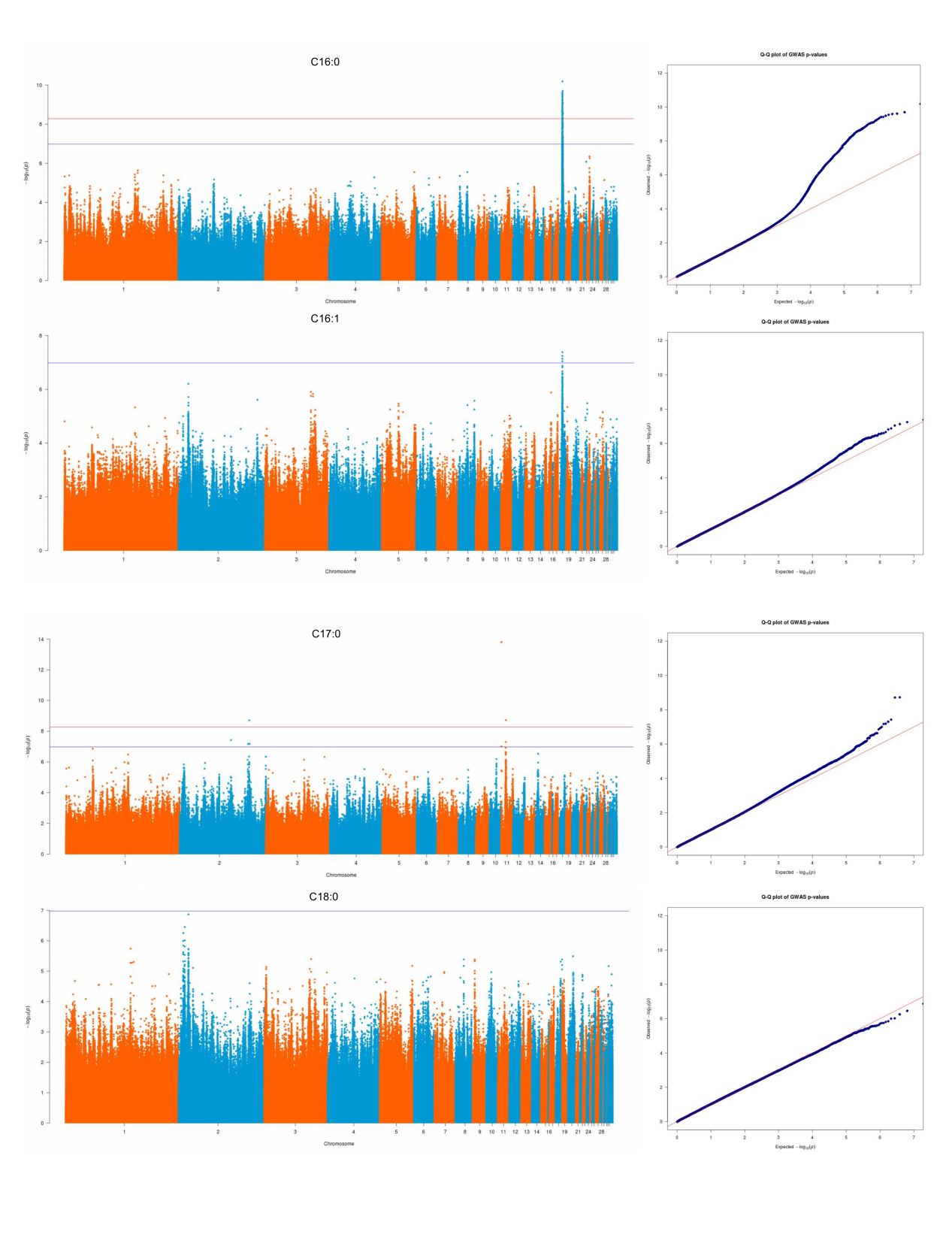
**

**
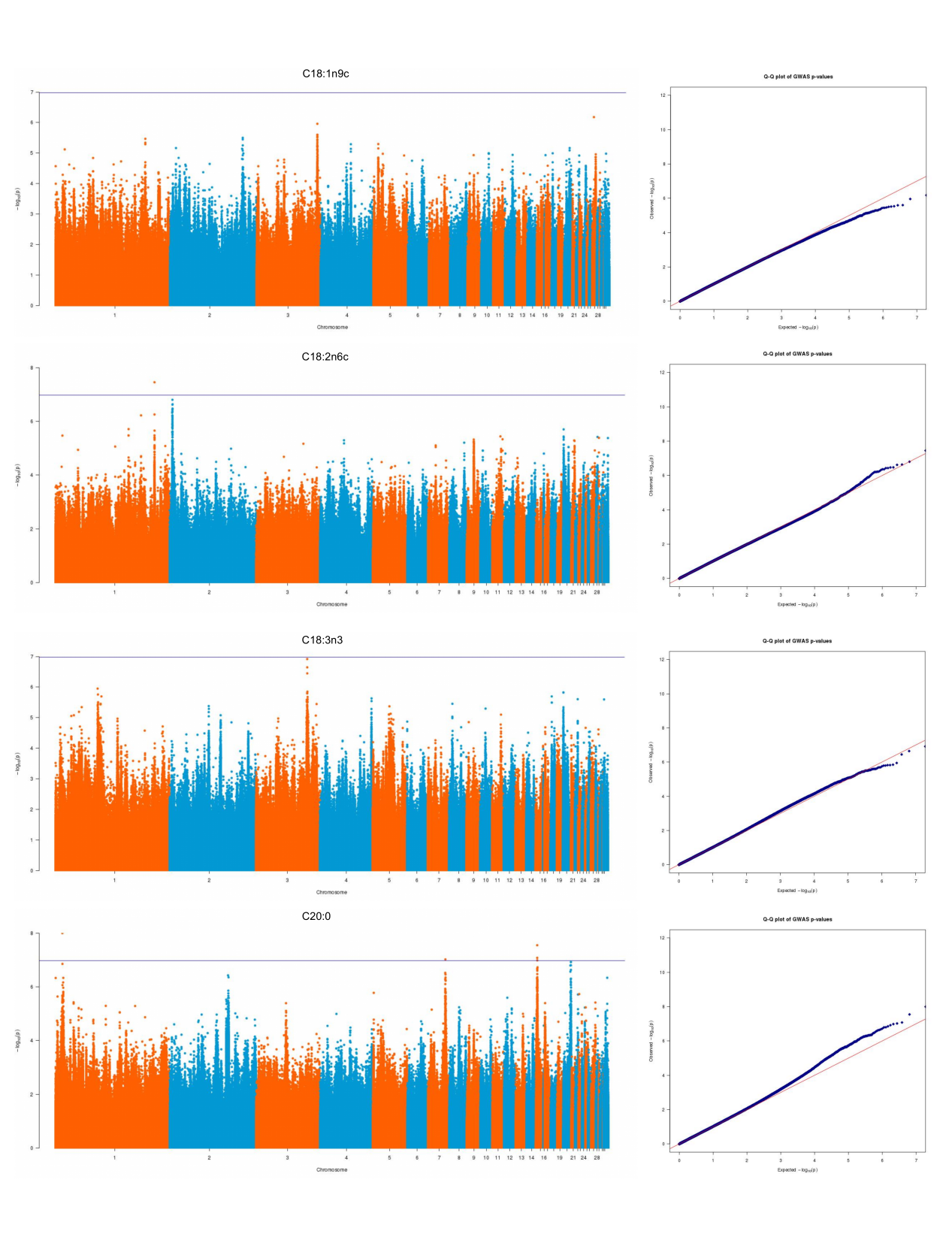
**

**
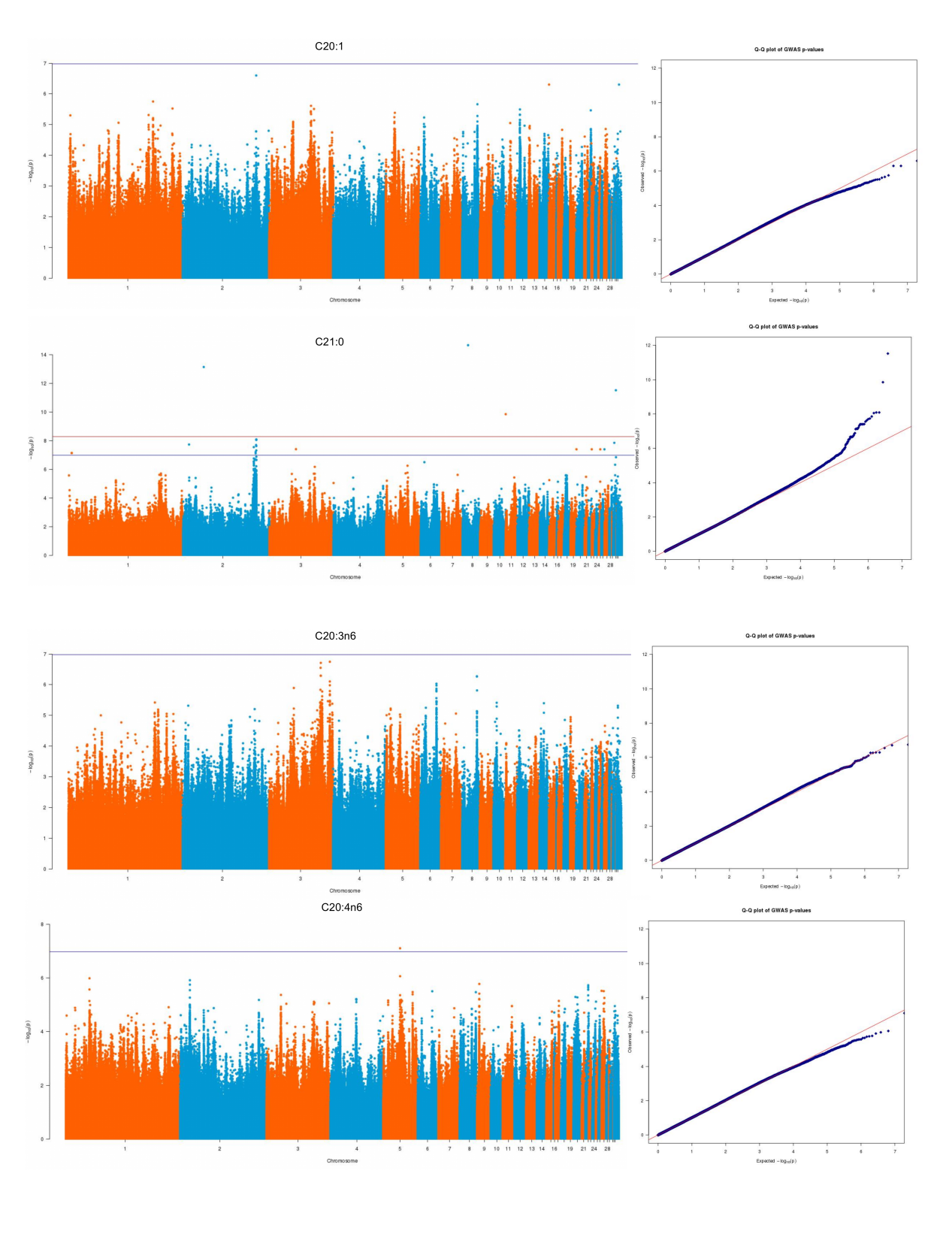
**

**
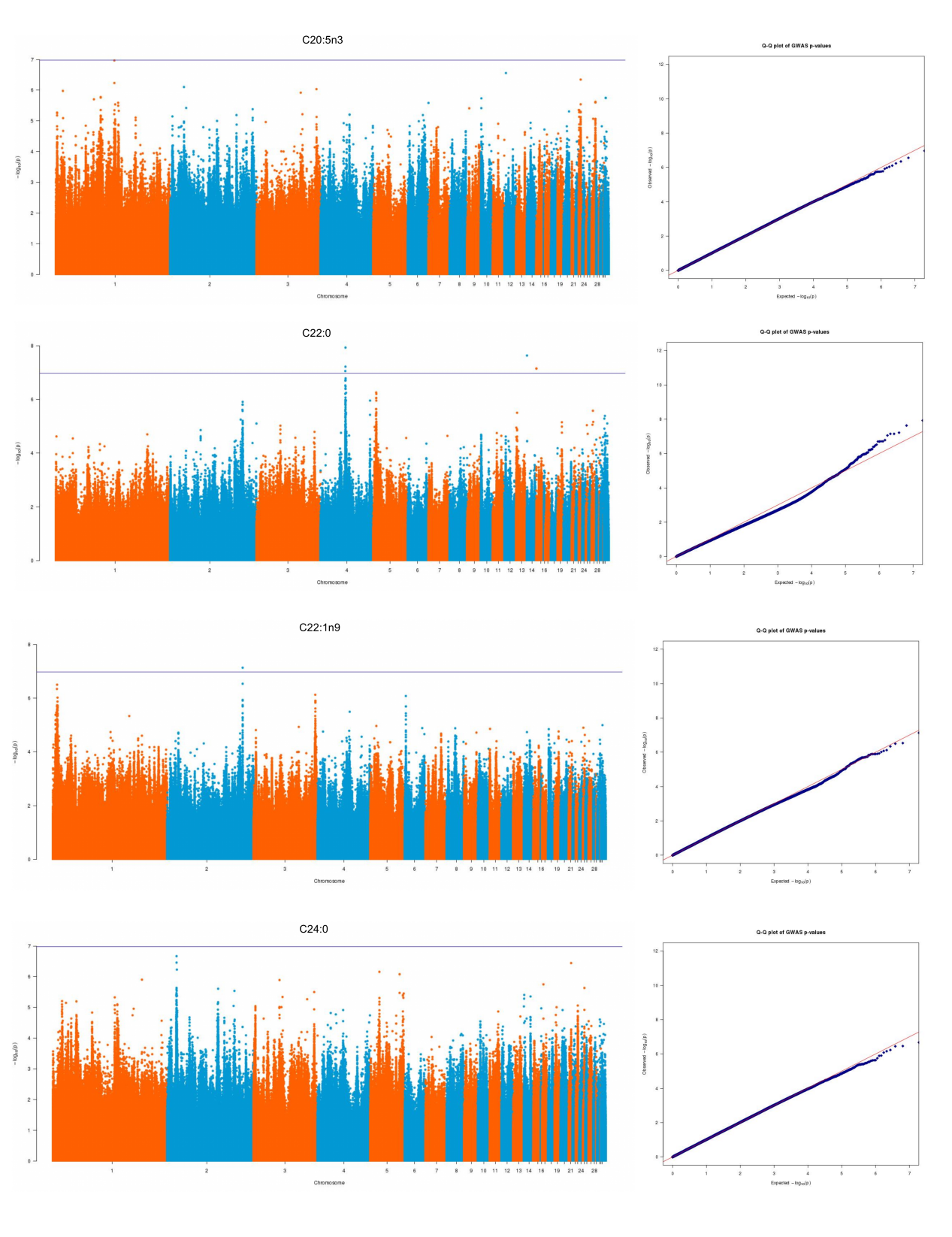
**

**
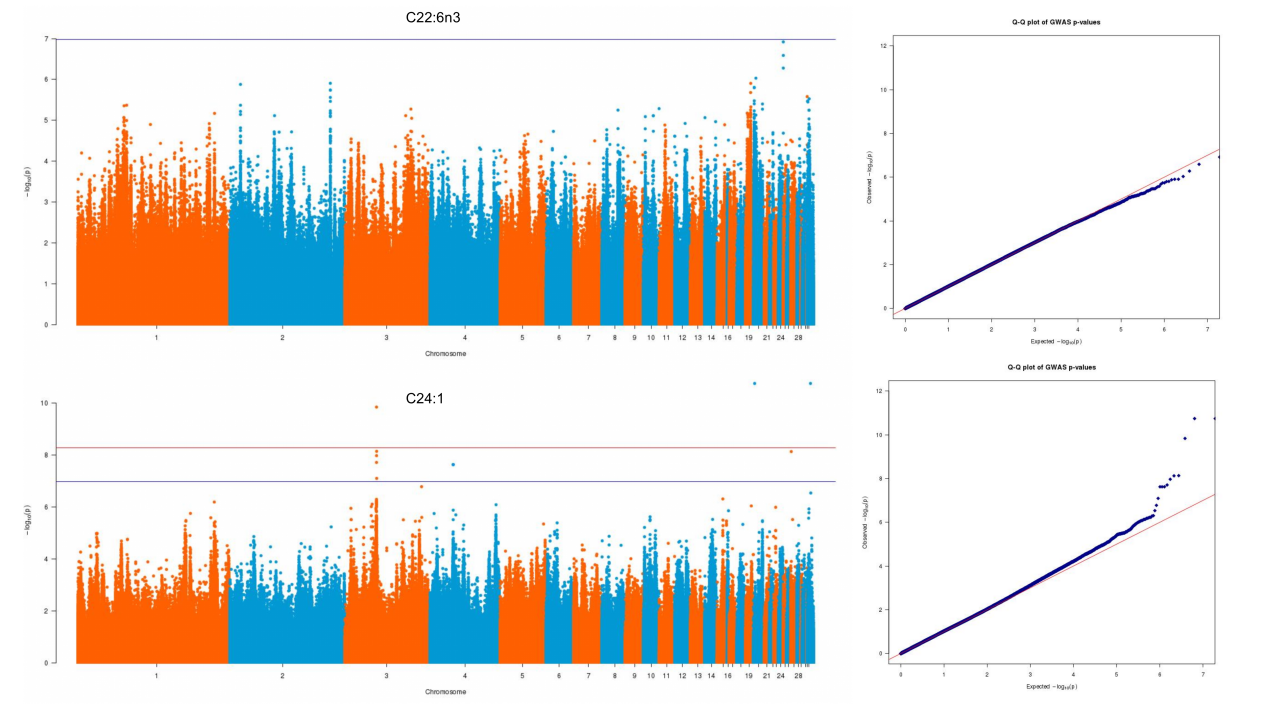
**

**Figure S3:** The manhattan and Q-Q plot of the GWAS analysis associated with the content of FAs content in breast muscle tissue (n=516).

**
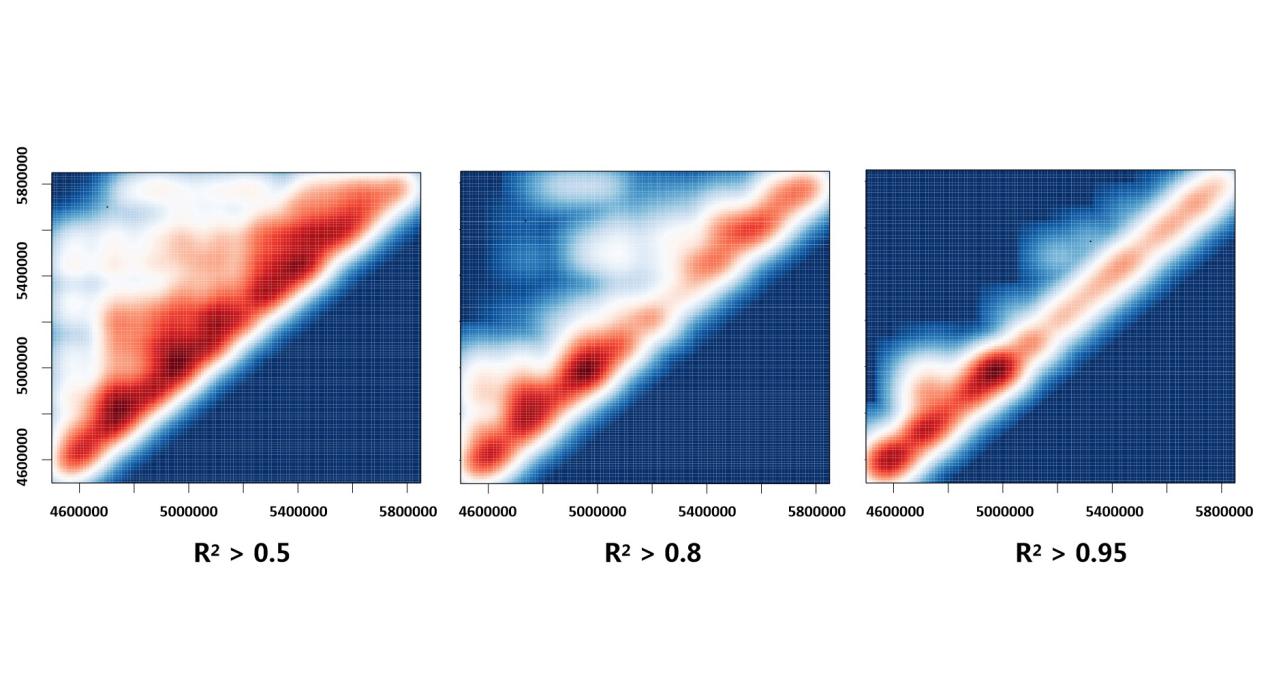
**

**Figure S4:** The linkage disequilibrium (LD) analysis of 14,938 SNPs in the region of 1.251 Mb (chr18:4,548,962-5,800,023) with the suggestive threshold line ±20 kb.

**
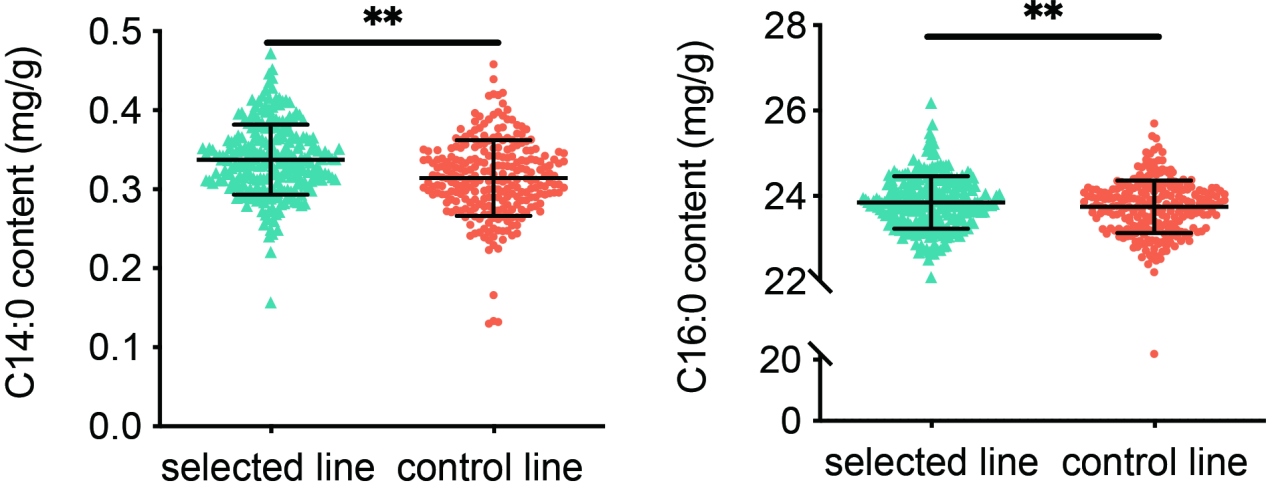
**

**Figure S5:** The comparison of C14:0 and C16:0 contents in the breast muscle tissue between the selected population and controls (n=516). **p* < 0.05, ***p* < 0.01, ****p* < 0.001.

**
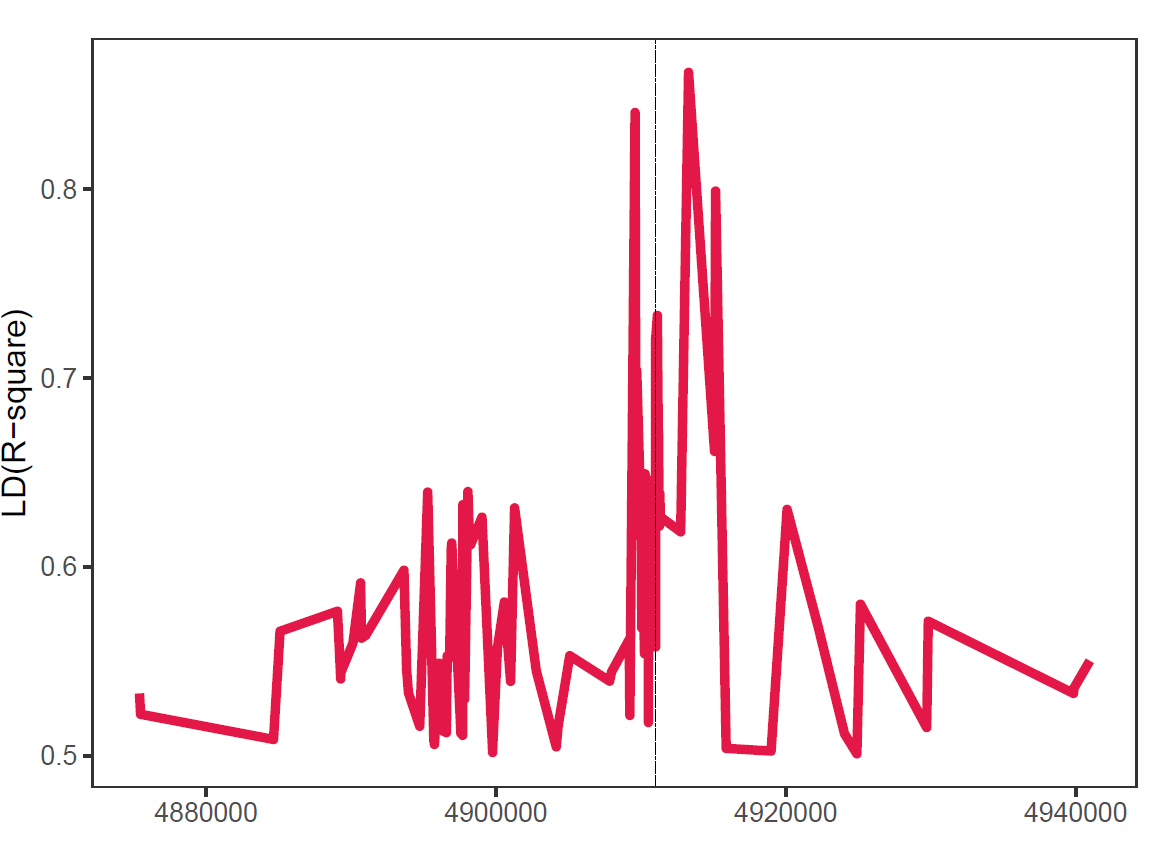
**

**Figure S6:** The linkage disequilibrium (LD) analysis of the linked SNP (chr18:4910969, [rs315349829](https://asia.ensembl.org/Gallus_gallus/Variation/Summary?db=core;r=18:4910969-4911000;vf=20920877) [A/G]) and the SNP with the highest p-value (chr18:4910989, [rs312](https://asia.ensembl.org/Gallus_gallus/Variation/Summary?db=core;r=18:4910969-4911000;vf=20920877)544499 [G/A]) at a distance of 20 bp.

**
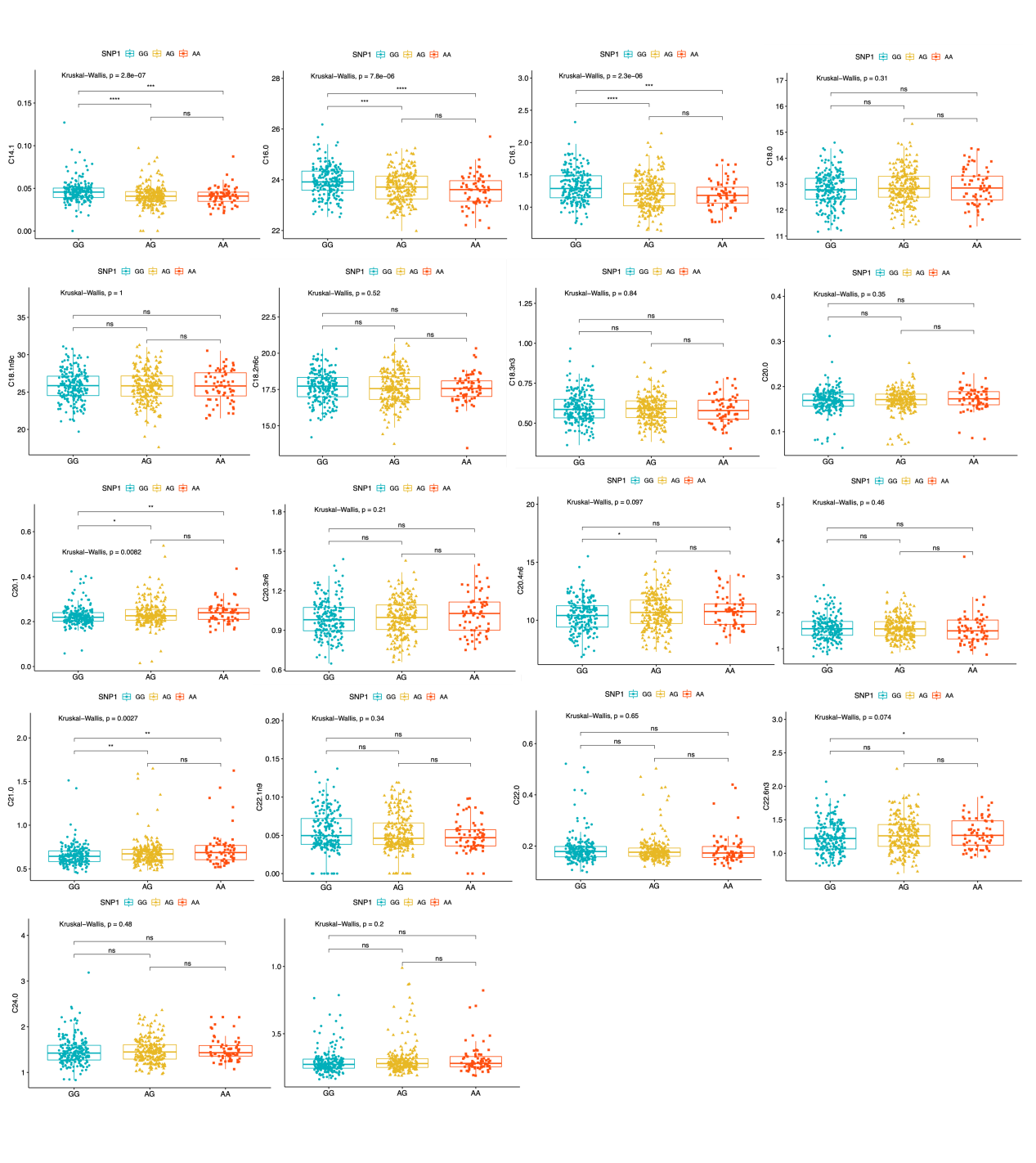
**

**Figure S7:** The effect of the [GG] and [AA] homozygous genotypes homozygotes in the ref-genotype rs315349829 on the FA content in breast muscle tissue, except for C14:0 and C16:0 (n=516).

**
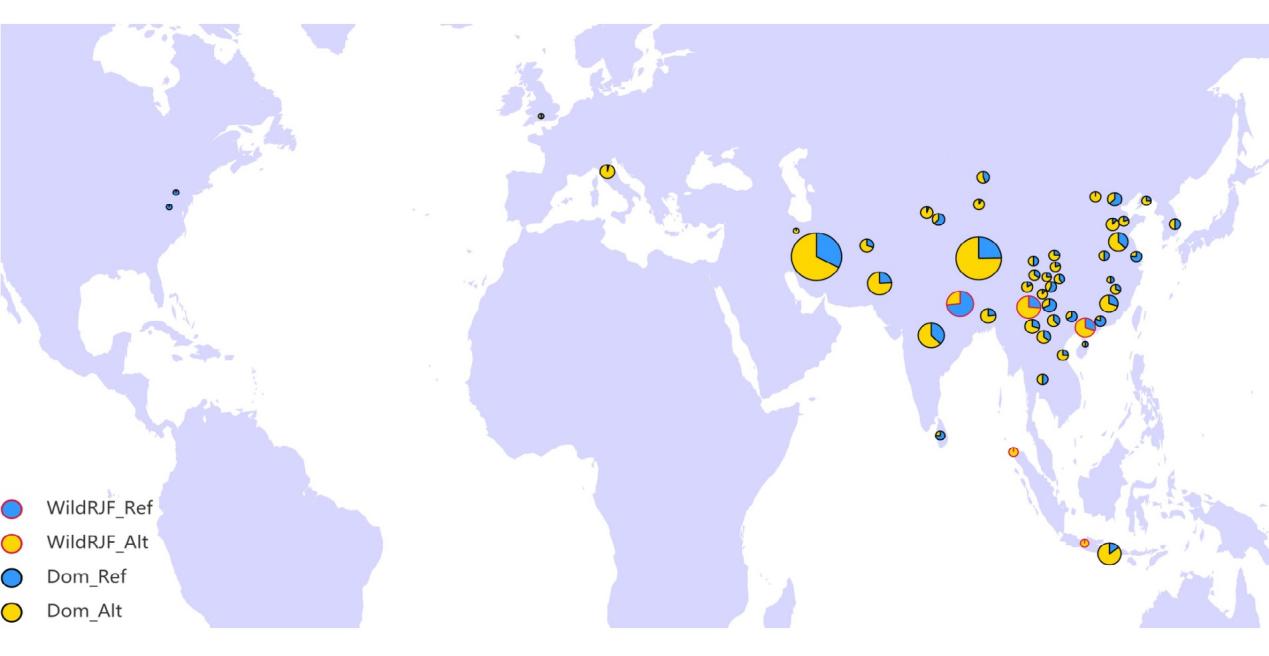
**

**Figure S8:** The widespread distribution of the SNP [rs315349829](https://asia.ensembl.org/Gallus_gallus/Variation/Summary?db=core;r=18:4910969-4911000;vf=20920877) of [A] and [G] mutations in multi-breeds from all over the world.

**
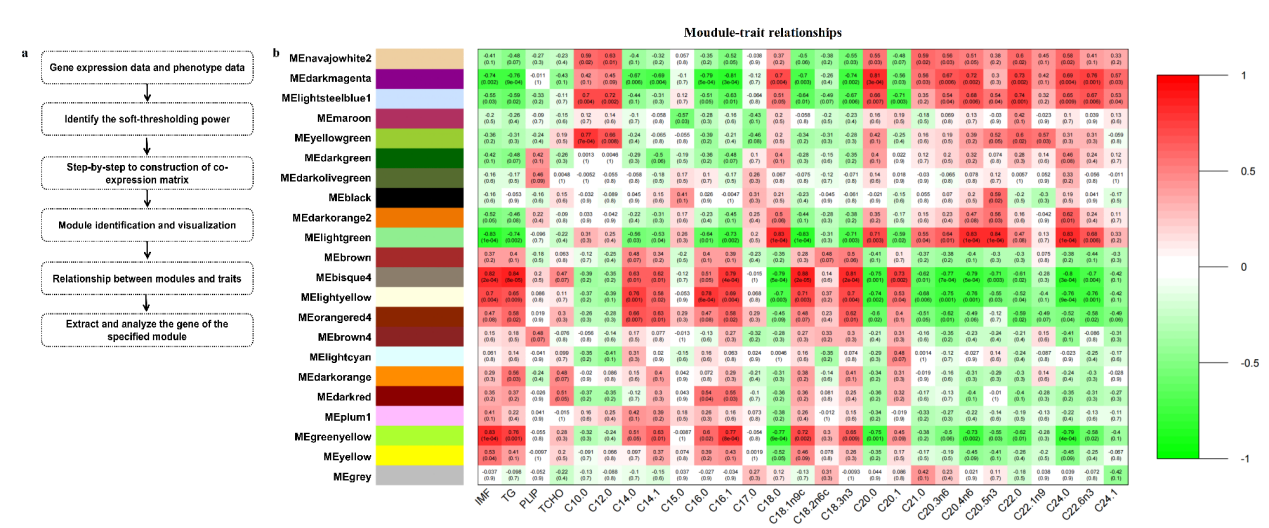
**

**Figure S9:** The weighted gene co-expression network analysis (WGCNA) on the 14,259 expressed genes (n =16) in chicken breast muscle tissue by transcriptome and phenotypic data (including IMF, TGs and FAs). **a.** Analysis process of WGCNA. **b.** Relationship between gene modules and traits in chicken breast muscle tissue (red means positive correlation, and green means negative correlation).

**
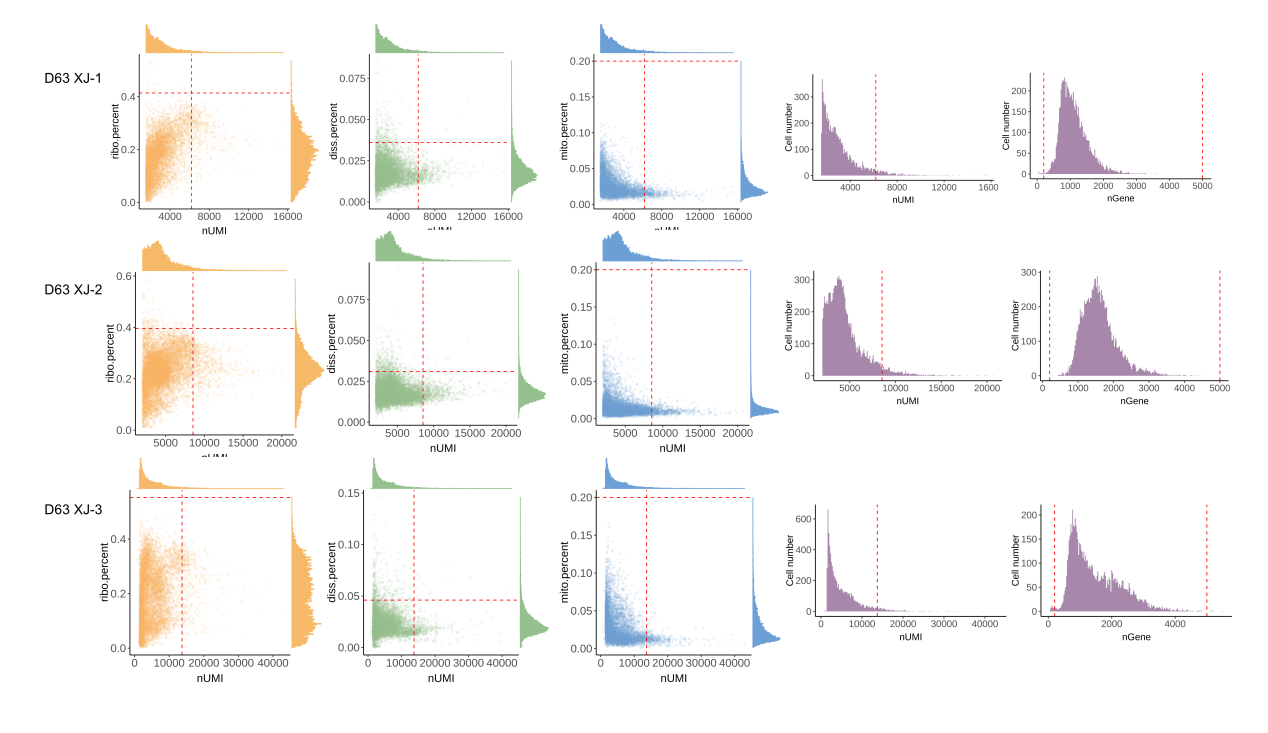
**

**Figure S10:** The experimental operation process and data quality control of the single-cell RNA sequencing in this study. **a.** The experimental operation process using the breast muscle samples at the age of 63 days. **b.** The data quality control of ribo.percent and cell number (n=3).

**
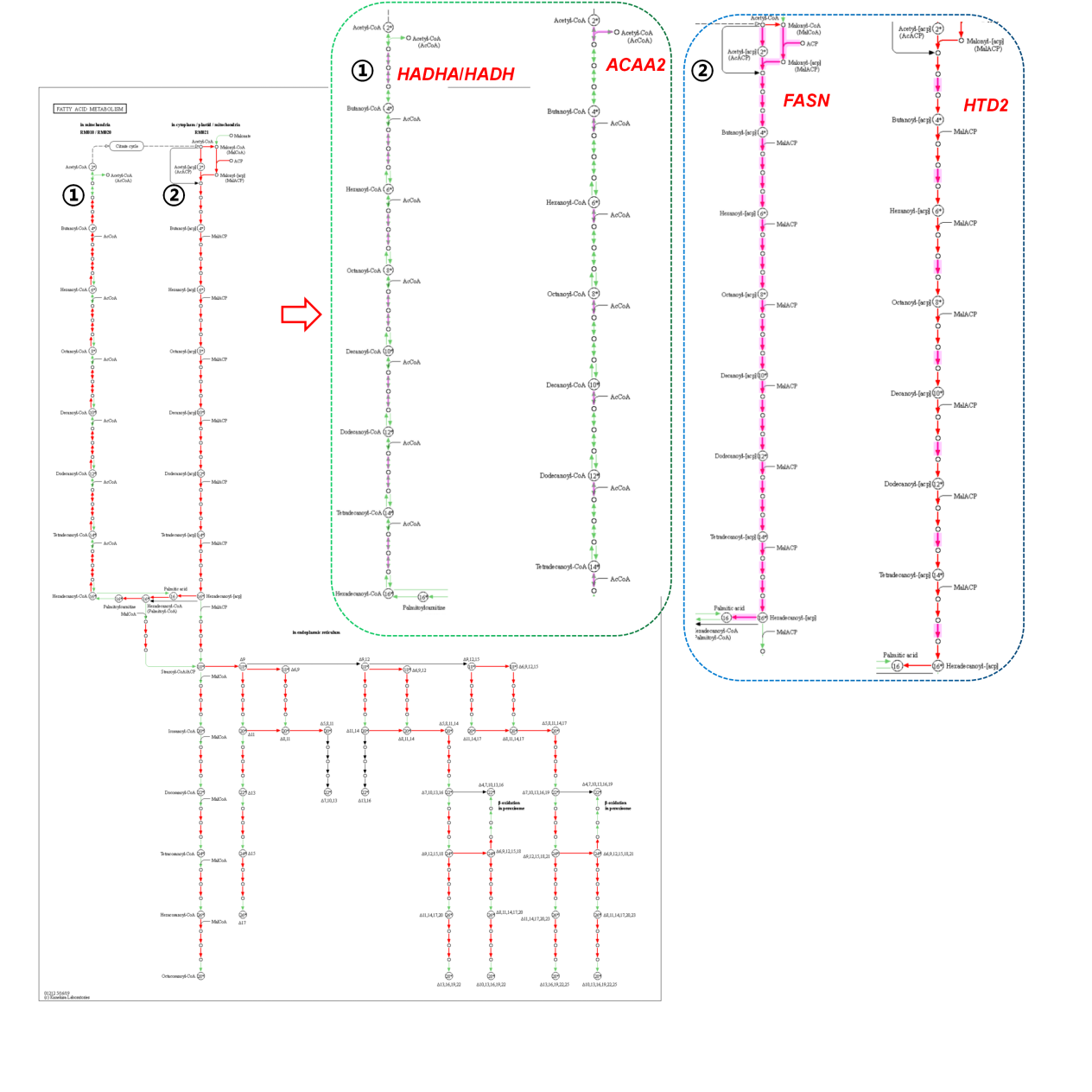
**

**Figure S11:** The enriched FA metabolism pathway based on 3,483 DEGs between myocytes and adipocytes in breast muscle tissue by single-cell RNA sequencing. FASN is the key enzyme in the *de novo* synthesis of FAs, using the *acetyl*-CoA or malonyl-CoA from the oxidative degradation of FA as substrate.

**Supplementary Tables (see the excel files)**

**Table S1.** The primers of *FASN* and *ACTB* gene used for qPCR in this study.

**Table S2.** Selective sweep windows based on fixed index (*F_ST_*) method. Chromosome region information for the top 1% threshold of *F_ST_* were retained.

**Table S3.** The genes annotated by the chromosom region of the top 1% F_ST_ value of fixed index (*F_ST_*). All the sweep windows were annotated based on the reference genome GRCg6a.

**Table S4.** The enriched KEGG pathways based on 845 genes annotated in chromosome regions with the top 1% *F_ST_* value.

**Table S5.** The content of IMF for GWAS.

**Table S6.** The top 1% SNPs (n=98,869) information of GWAS results for IMF trait.

**Table S7.** Genes annotated with top 1% SNPs information for GWAS results of IMF trait.

**Table S8.** The content of fatty acids for GWAS.

**Table S9.** The identified 14,938 SNPs in the chr18:4,548,962-5,800,023 region with the suggestive threshold line±20 Kb.

**Table S10.** The genetic selection signals for single site of candidate regions *F_ST_* (standard value of 0.1) and Pi.

**Table S11.** Proportion of phenotypic variance explained (PVE) by SNP (rs315349829) in JXY chicken.

**Table S12.** Data of myristic acid content and genotype of individuals carrying the variation (rs315349829).

**Table S13.** The genetic correlation of SNPs (rs315349829) with myristic acid content in breast tissue of multiple Chinese native chicken populations.

**Table S14.** Phenotypic data for WGCNA analysis (including IMF, TG and FAs).

**Table S15.** The information of genes enriched in the ME light yellow module.

**Table S16.** Quality control metrics for all simples scRNA-seq data sets.

**Table S17.** The cell activity in three chicken breast muscle samples at the age of 63 days.

**Table S18.** The screened 3483 DEGs (|log2 FC| ≥ 0.585) in myocytes *vs*. adipocytes.
